# Systematic Review and Expert Consensus on the Use of Long-acting Monoclonal Antibodies for Prevention of Respiratory Syncytial Virus Disease: ARMADA (Advancing RSV Management And Disease Awareness) Taskforce

**DOI:** 10.1093/ofid/ofaf396

**Published:** 2025-07-02

**Authors:** Paolo Manzoni, Eugenio Baraldi, Fabio Midulla, Olivier Claris, Sandro Dessardo, Terho Heikkinen, Richard Thwaites, Bosco Paes, Xavier Carbonell-Estrany, Dmytro Dobryanskyy, Merih Cetinkaya, Adel S Al Harbi, Ji-Man Kang, Anne Goh Eng Neo, Hsin Chi, Guilherme Sant’Anna, Mónica Villa Guillén, Gonzalo Luis Mariani, Marco Aurelio Palazzi Safadi, Soledad Urzua, Heather J Zar, Pierre Goussard, Barry Rodgers-Gray, Nicola Waghorne, Manuel Sanchez Luna

**Affiliations:** Department of Public Health and Pediatric Sciences, University of Torino School of Medicine, Turin, Piedmont, Italy; Division of Paediatrics and Neonatology, Degli Infermi Hospital, Ponderano, Italy; Department of Women's and Children's Health, University Hospital of Padova, Veneto, Italy; Institute of Pediatric Research, “Città della Speranza”, Padova, Veneto, Italy; Department of Pediatrics and Pediatric Neuropsychiatry, Sapienza University of Rome, Rome, Italy; Hospices Civils de Lyon, Hôpital de la Croix Rousse, Service de néonatologie et réanimation néonatale, Bron, France; EA 4129, Université Claude Bernard Lyon 1, Lyon, France; Department of Pediatrics, University Hospital Centre, Zagreb, Croatia; Department of Pediatrics, University of Turku and Turku University Hospital, Turku, Finland; The Neonatal Unit, Royal Stoke University Hospitals, Stoke-on-Trent, UK; Department of Pediatrics (Neonatal Division), McMaster University and McMaster Children's Hospital, Hamilton, Ontario, Canada; Neonatology Service, Hospital Clinic, Barcelona, Spain; Department of Pediatrics, Lviv National Medical University, Lviv, Ukraine; Department of Neonatology, Health Sciences University, Basaksehir Cam and Sakura City Hospital, Istanbul, Turkey; Department of Pediatrics, Prince Sultan Military Medical City, Alfaisal University, Riyadh, Saudi Arabia; Department of Pediatrics, Severance Children's Hospital, Yonsei University College of Medicine, Seoul, South Korea; Institute for Immunology and Immunological Diseases, Yonsei University College of Medicine, Seoul, South Korea; Department of Pediatrics, KK Women's and Children's Hospital, Singapore; Department of Pediatrics, MacKay Children's Hospital, Taipei, Taiwan; Department of Pediatrics, MacKay Memorial Hospital, Taipei, Taiwan; Department of Pediatrics, McGill University Health Centre, Montreal, Quebec, Canada; Department of Medical Management, National Institute of Health Children´s Hospital of Mexico Federico Gómez, Mexico City, Mexico; President of the National Federation of Neonatology of Mexico, Mexico City, Mexico; Division of Neonatology, Departments of Pediatrics, Instituto Universitario Hospital Italiano, Buenos Aires, Argentina; Department of Pediatrics, Santa Casa de São Paulo School of Medical Sciences, São Paulo, Brazil; Department of Neonatology, School of Medicine, Pontificia Universidad Catolica de Chile, Santiago, Chile; Department of Paediatrics and Child Health, Red Cross War Memorial Children's Hospital, University of Cape Town, Cape Town, South Africa; SA Medical Research Council Unit on Child and Adolescent Health, University of Cape Town, Cape Town, South Africa; Department of Paediatrics and Child Heath, Stellenbosch University, Stellenbosch, South Africa; Paediatric Pulmonology and Paediatric Intensive Care, Tygerberg Hospital, Parow, South Africa; Violicom Medical Limited, Aldermaston, UK; Violicom Medical Limited, Aldermaston, UK; Neonatology Division, University General Hospital Gregorio Maranon, Complutense University of Madrid, Madrid, Spain

**Keywords:** disease prevention, long-acting monoclonal antibodies (LAmAbs), public health impact, respiratory syncytial virus (RSV)

## Abstract

**Background:**

Long-acting monoclonal antibodies (LAmAbs) could dramatically reduce the respiratory syncytial virus (RSV) disease burden in children if implemented using clear, evidence-based recommendations.

**Methods:**

The ARMADA Taskforce—an international, multidisciplinary expert panel—undertook a systematic review to develop LAmAbs consensus recommendations for RSV disease prevention in children.

**Results:**

The Taskforce recommends LAmAbs for all infants aged <8 months in the absence of maternal RSV vaccination, preterm infants (<37 weeks’ gestational age) aged <12 months, and children <24 months with high-risk conditions. Seasonal LAmAb administration is recommended, although in RSV-endemic countries decisions should be made locally concerning administration year-round or with peak RSV incidences.

**Conclusions:**

The Taskforce strongly endorses LAmAbs implementation based on their efficacy, effectiveness, and public health impact. These recommendations provide a blueprint to inform guidelines worldwide. Wider equitable access to LAmAbs at affordable prices, especially in low- and middle-income countries is needed to reduce the childhood RSV burden.

## Teaser

The ARMADA Taskforce, representing 18 countries, has developed clear, evidence-based recommendations on the use of long-acting monoclonal antibodies for RSV disease prevention that can provide a blueprint to inform the development of regional and national society guidelines across the world.

## RESEARCH IN CONTEXT

### Evidence Before This Study

Prevention of respiratory syncytial virus (RSV) disease, a prominent cause of bronchiolitis and pneumonia in infants and children, has relied on the monoclonal antibody palivizumab over recent decades, with its use restricted to high-risk populations, such as those born prematurely (≤35 weeks’ gestational age) or with comorbidities. Newer long-acting monoclonal antibodies (LAmAbs) could significantly reduce the burden of RSV disease in all children, but evidence-based recommendations to guide their use around the world are needed to maximize their benefits. The ARMADA (Advancing RSV Management And Disease Awareness) Taskforce systematically searched PubMed, Embase, the Cochrane Library, and the gray literature from inception to February 2024 using keywords relating to RSV and LAmAbs to identify evidence supporting LAmAbs for RSV disease prevention. Of 2145 citations screened, 81 reported clinical trial data, real-world evidence, guidelines, or cost-analyses in preterm and term infants without comorbidities, special populations, including bronchopulmonary dysplasia or chronic lung disease, congenital heart disease, and other high-risk groups. The evidence demonstrated that LAmAbs are highly efficacious and effective at preventing RSV disease, while being well-tolerated and cost-effective in both high and lower-middle income countries (LMIC), although only at prices less than USD $5 per immunization in the latter.

### Added Value of This Study

Predicated on this systematic evaluation of the existing evidence, current national guidelines, and expert experiences, the ARMADA Taskforce recommends the use of LAmAbs for all infants aged <8 months in the absence of maternal RSV vaccination, preterm infants (<37 weeks’ gestational age) aged <12 months, and children <24 months with high-risk conditions (eg, chronic lung disease, congenital heart disease) at the start of or during the RSV season. Seasonal LAmAb administration is recommended, although in countries where RSV is endemic a decision should be made locally concerning administration throughout the year or to coincide with annual peak RSV incidences. This evidence-based consensus provides a universal template to inform the development of regional and national society guidelines for the use of LAmAbs to prevent severe RSV disease in infants and children across the world.

### Implications of All the Available Evidence

The ARMADA Taskforce strongly endorse the global implementation of LAmAb programs to prevent RSV disease in infants and young children, while recognizing affordability is a challenge in LMICs. Product access in these countries is crucial to reduce global inequity and the universal burden of severe RSV disease; therefore, strong collaboration between stakeholders, distributors, funders, and public health programs will be central to successful implementation and should be prioritized. To further maximize the use of LAmAbs, future research should focus on their effectiveness in children with underlying medical conditions, postimplementation surveillance for RSV disease through 2 years of age, their impact on long-term respiratory morbidity and non-RSV outcomes (eg, all cause lower respiratory tract infection, otitis media, antibiotic prescription) as well as their concurrent use with maternal RSV vaccine.

## INTRODUCTION

RSV is the leading viral cause of bronchiolitis and childhood pneumonia and is estimated to result in approximately 33 million lower respiratory tract infections (LRTIs), 3.6 million related hospitalizations (RSVH), and >101 000 deaths annually in children aged <5 years worldwide [1]. RSV is also responsible for 10%–20% of medically attended infant respiratory infections, a term typically used to refer to infections requiring solely outpatient care, visits to the emergency department, and/or hospitalization [2]. Early life RSV-LRTI has also been associated with long-term respiratory morbidity, including recurrent respiratory infections, recurrent wheezing, asthma, and impaired lung function [3]. Until recently, prevention of RSV disease in infants relied on the monoclonal antibody palivizumab, administered as a monthly immunization to only the most high-risk infants, specifically those born prematurely (<35 weeks’ gestational age [wGA]), or with bronchopulmonary dysplasia (BPD)/chronic lung disease (CLD) or congenital heart disease (CHD) [4, 5]. However, risk factors for severe RSV-LRTI are not present in the majority of infants who experience RSVH [6, 7]. The introduction of LAmAbs (such as nirsevimab and, in the near future, clesrovimab) has the potential to dramatically reduce the intensity of RSV epidemic waves and concomitant capacity surges on pediatric acute care systems and thereby profoundly impact the global burden of RSV disease.

To maximize the benefits of LAmAbs, it is essential that their deployment is guided by clear, evidence-based recommendations. The ARMADA Taskforce was formed with the aim of developing an expert- and evidence-driven consensus on LAmAbs for RSV disease prevention. It is anticipated that the consensus recommendations will provide a universal template or blueprint to inform the development of regional and national society guidelines across the world.

## METHODS

### ARMADA Taskforce

The ARMADA Taskforce is an international, multidisciplinary panel of pediatric infectious disease specialists, neonatologists, pediatric pulmonologists, and recognized RSV experts, who were invited by P.M., based on their expertise, to form a consensus panel.

### Remit of Consensus

Evidence was reviewed for the following key areas:

- LAmAb use in preterm and term infants without comorbidities
- LAmAb use in special populations, including BPD/CLD or CHD, and other high-risk groups
- Cost-effectiveness of LAmAbs
- Current guidelines for LAmAbs

### Identification of Evidence

A systematic literature review (SLR) was overseen by P.M. and conducted by 2 experienced reviewers (B.R.-G. and N.W.) to address the research question: *What is the evidence to support long-acting monoclonal antibodies for RSV disease prevention?* For the purpose of this SLR, LAmAb was defined as an agent with a sufficiently long half-life that provides protection against RSV disease via the administration of a single dose that is effective for the duration of a single RSV season [8]. Systematic methods were used to identify and appraise relevant research, and to analyze and report data from the included studies according to the PRISMA guidelines [9]. The protocol was registered in PROSPERO: CRD42024517044 (Supplementary File 1) [10]. PubMed (Medline), Embase, and the Cochrane Library were searched from database inception until 23 February 2024, using keywords relating to RSV and LAmAbs. To increase the robustness of the review, the gray literature [11] was also assessed to capture a wider range of sources, including government reports and conference abstracts. Identified studies were evaluated by 2 independent reviewers against predefined PICOS (Population, Intervention, Comparator, Outcomes, Study design) criteria using a 2-phase approach: (1) titles/abstracts and (2) eligible full texts. Data were extracted from the full text of all included articles by 1 reviewer, and quality checked by a second reviewer. Additional studies meeting the PICOS criteria identified by the authors during the development of this paper were also included to ensure that the consensus was as up-to-date as possible. Risk of bias was assessed using the Cochrane Collaboration Risk of Bias 2 tool [12] for randomized controlled trials (RCT), the RTI Item Bank [13] for observational studies and the Quality of Health Economic Studies List [14] for cost-effectiveness analyses.

### Evaluation of Evidence and Recommendations

The consensus recommendations were developed as follows. First, the ARMADA Taskforce agreed on a framework, upon which P. M. drafted recommendations. The recommendations were reviewed and edited by the Taskforce, which then voted on each recommendation (1 = fully agree; 2 = partially agree; 3 = undecided; 4 = disagree; 5 = strongly disagree), with consensus defined as ≥75% of the Taskforce voting as “fully agree” or “partially agree.” The strength of evidence for each recommendation was rated according to the Oxford Centre for Evidence-Based Medicine Levels of Evidence [15] and Grading of Recommendations Assessment, Development and Evaluation (GRADE) [16].

## EVIDENCE FOR THE USE OF LAMABS FOR RSV INFECTION

### Systematic Review

**Figure 1.**
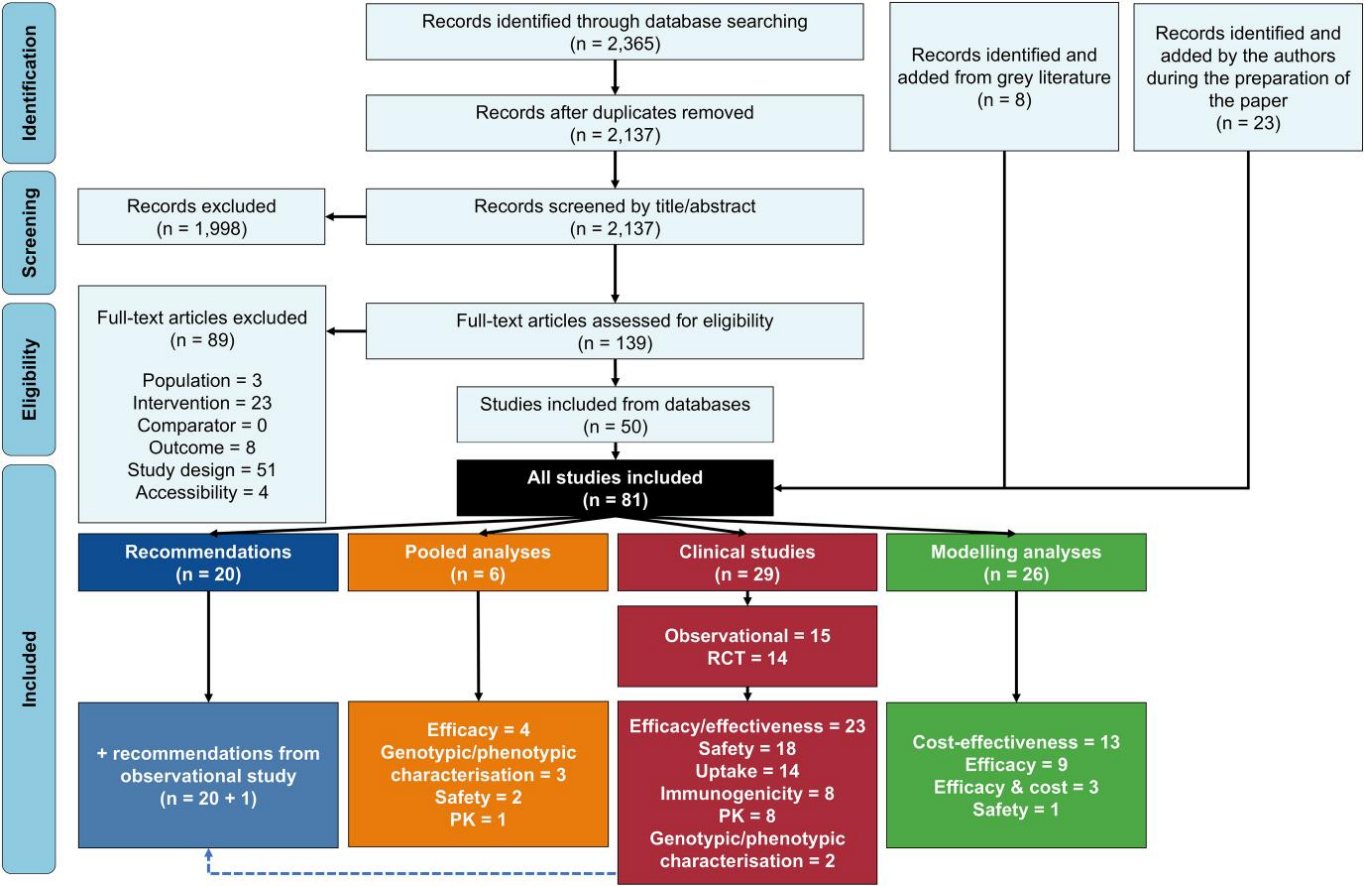
PRISMA diagram. Evidence for the use of LAmAbs in RSV infection. Abbreviations: LAmAbs, long-acting monoclonal antibodies; PK, pharmacokinetics; RCT, randomized controlled trial; RSV, respiratory syncytial virus.

The SLR identified 2145 citations of which 58 met the inclusion criteria, with a further 23 included by the authors during the preparation of the manuscript (up to December 2024), resulting in a total of 81 meeting the PICOS criteria (Figure 1 and Supplementary File 2): 29 clinical studies [17–45], 6 pooled analyses [46–51], and 26 modeling analyses [52–77] that included 13 cost-effectiveness studies [52–64] and 20 recommendations/guidelines [78–97].

### Evidence for LAmAbs in Healthy Preterm and Term Infants Without Comorbidities

In meta-analyses, nirsevimab reduced medically attended RSV-LRTI and RSVH by 74%–80% and 75%–88%, respectively (Table 1) [47, 48, 50, 51]. The nirsevimab data used in these meta-analyses were primarily derived from 2 global, placebo-controlled RCTs (phase 2b trial [24] of infants 29^0/7^ through 34^6/7^ wGA and the phase 3 MELODY trial [23] [primary cohort, ≥35 wGA]), which reported a reduction in medically attended RSV-LRTI by 70.1% (95% confidence interval [CI], 52.3–81.2; *P* < .001) and 74.5% (95% CI, 49.6–87.1; *P* < .001), respectively, and RSVH by 78.4% (95% CI, 51.9–90.3; *P* < .001) and 62.1% (95% CI, −8.68 to 6.8; *P* = .07), respectively. However, the phase 3 MELODY (primary cohort) trial [23] was impacted by coronavirus disease 2019 with substantially fewer RSVHs than originally projected. Later analysis of the full cohort reported a reduction in RSVH of 76.8% (95% CI, 49.4–89.4) [21]. Efficacy against medically attended RSV-LRTI (76.4%; 95% CI, 62.3–85.2) was consistent with that identified in the primary cohort [21]. The highest efficacy of nirsevimab 83.2% (95% CI, 67.8–92.0; *P* < .001) against RSVH was reported in the phase 3b HARMONIE open-label, randomized trial for infants born at ≥29 wGA entering their first RSV season in France, Germany, and the United Kingdom [17].

**Table 1.** Efficacy of LAmAbs in Meta-analyses

				Nirsevimab Efficacy Versus Placebo			
Citation	Design	Study Population	Sample Size	Medically Attended RSV-LRTI	RSVH	All-cause Medically Attended LRTI	All-cause Respiratory Hospital Admission
Sun et al. 2023 [47]	Random effects network meta-analysis	Healthy preterm and term infants: Phase 2b trial [23] Phase 3 MELODY trial (primary cohort) [24]	Nirsevimab: n = 1963 Placebo: n = 980	76% reduction	75% reduction	NR	NR
Simões et al. 2023 [48]	Prespecified pooled analysis of 2 RCTs	Healthy preterm and term infants: Phase 2b trial [23] (excludes infants ≥5 kg who did not receive the recommended weight-banded dose of 100 mg) Phase 3 MELODY trial (primary cohort) [24]	Nirsevimab: n = 1564 Placebo: n = 786	79.5% reduction	77.3% reduction	35.4% reduction	43.8% reduction
Turalde-Mapili et al. 2023 [50]	Random effects meta-analysis	Healthy preterm and term infants: Phase 2b trial [23] Phase 3 MELODY trial (primary cohort) [24]	Nirsevimab: n = 1963 Placebo: n = 980	74% reduction	76% reduction	NR	NR
Ricco et al. 2024 [51]	Random effects meta-analysis	Healthy preterm and term infants from RCTs and real-world studies, with the latter also including children with comorbidities	Nirsevimab: n = 33 884 (RCTs: n = 7582 Real-world studies: n = 26 302) Placebo n = 9365	NR	88.4% reduction	NR	NR

Shaded areas represent statistically significant (*P* < .05) difference versus placebo.

Abbreviations: LAmAb, long-acting monoclonal antibody; LRTI, lower respiratory tract infection; NR, not reported; RCT, randomized controlled trial; RSV, respiratory syncytial virus; RSVH, RSV hospitalization.

A recent post hoc analysis of the MELODY trial found that nirsevimab protected against both single RSV infections and co-infections and, importantly, there was no evidence of replacement of RSV by other respiratory viruses [45]. A separate analysis of healthy infants in the MELODY trial reported that the incidence of medically attended RSV-LRTI in the second RSV season was low (nirsevimab: 0.7%; placebo: 0.4%) with no RSVH, thus providing no evidence to support antibody dependent enhancement in nirsevimab recipients [27]. Pooled analysis (phase 2b and MELODY trials) reported a lack of nirsevimab resistance (>99% of RSV F protein sequences remained susceptible) and showed sustained, high levels of RSV neutralizing antibodies (>50-fold higher than baseline) at 150 days postdose in term and preterm infants; further supporting the efficacy and neutralization activity of nirsevimab against both RSV A and B strains throughout the RSV season [18, 20, 49]. Additionally, several studies have shown nirsevimab to be well-tolerated with similar rates of adverse events (AE) and serious AE as placebo and/or palivizumab [17, 19, 21–25].

Data on a new LAmAb, clesrovimab, are more limited. Preliminary results have recently emerged, but full clinical trial publications are awaited. A phase 2b/3 study in healthy preterm and full-term infants identified a 60.4% (95% CI, 44.1–71.9; *P* < .001) reduction in medically attended RSV-LRTI and 84.2% reduction in RSVH (95% CI, 66.6–92.6; *P* < .001) for clesrovimab versus placebo [43]. In a phase 3 study of infants and children at increased risk of severe RSV disease (prematurity ≤35 wGA, CLD, CHD), comparable rates of medically attended RSV-LRTI (3.6% [95% CI, 2.0–6.0] vs 3.0% [95% CI, 1.6–5.3]) and RSVH (1.3% [95% CI, 0.4–3.0] vs 1.5% [95% CI, 0.3–3.3]) were reported for 1 dose of clesrovimab versus monthly palivizumab, respectively [44]. Data from both studies [43, 44], as well as from a phase 1b/2a study [26], suggest that clesrovimab is well-tolerated with a similar safety profile to placebo and palivizumab.

Evidence from the clinical studies was deemed high quality (19 had low risk of bias [17–19, 21–26, 28–30, 33–37, 39, 40], 4 had some methodological concerns [20, 37, 41, 45], 3 could not be assessed because only abstracts [27, 31] or summary reports [32] were available (Supplementary File 3), albeit primarily reflecting LAmAb use in high-income countries. However, the pivotal phase 2b trial did include 343 infants from 4 LMICs, whereas the phase 3 MELODY trial enrolled 463 infants from 2 LMICs in the primary cohort [23], rising to 745 infants from 6 LMICs in the full enrollment cohort [21]. In both trials, the LMIC population was predominantly from South Africa (n = 250 and n = 462, respectively). Further LAmAb evidence from LMICs, particularly demonstrating real-world effectiveness, is limited because of the inequity in availability, affordability, and implementation of RSV immunization in these countries. Nevertheless, within a modelling study, the effects of different nirsevimab administration approaches have been assessed in infants <6 months from 52 LMICs, albeit with efficacy assumptions derived from the phase 2b trial enrolling predominantly high-income country participants [73]. Assuming nirsevimab coverage similar to country-specific Bacillus Calmette-Guerin (BCG) and hepatitis B vaccine uptake, the median effectiveness using a year-round approach for averting RSVH was 58.1% (interquartile range 51.3–63.8), increasing to 66.2% (66.2–66.2) when assuming 100% coverage [73]. The median effectiveness of 4 seasonal approaches (administration in each epidemic month, or 1, 2, or 3 months prior) for averting RSVH ranged from 26.7 to 49.7%, increasing to 32.3–56.0% with 100% coverage; effectiveness improved with earlier administration before season onset [73].

### Evidence for LAmAbs in Other Specific High-risk Populations

Evidence for LAmAbs is more limited in infants traditionally considered at high-risk for severe RSV disease, such as those with CLD, CHD, and the immunocompromised. Within the phase 2/3 MEDLEY trial, which enrolled 310 infants with CHD/CLD and 615 infants ≤35 wGA entering their first RSV season, 7 infants had medically attended RSV-LRTI (4/616 infants [0.6%] receiving nirsevimab and 3/309 infants [1.0%] receiving palivizumab) [22]. Moreover, at day 151, serum levels of nirsevimab were similar between the preterm and CHD/CLD MEDLEY cohorts and akin to those reported in the MELODY trial [22]. Pharmacokinetic extrapolation of data from MEDLEY found nirsevimab exceeded the efficacy threshold (80%) for infants with CLD (94%), CHD (80%), and infants born <29 wGA (94%) [48]. Similarly, in 240 children with CHD/CLD who received 200 mg nirsevimab before entering their second RSV season, nirsevimab serum exposures were associated with efficacy rates achieved in healthy term and preterm infants (98% achieved target serum area under the curve) and no RSV-LRTI occurred through day 151 [19]. The antidrug antibody response was low and the safety profile of nirsevimab comparable to that of palivizumab in infants with CHD or CLD across both the first and second RSV seasons [19, 22]. Moreover, assuming efficacy similar to healthy infants in the MELODY trial, a study modelling the potential impact of nirsevimab in infants with CHD/CLD across both the first and second years of life, estimated that nirsevimab might prevent 60% of medically attended RSV-LRTI in these high-risk children through 24 months of age [69].

The MUSIC study, an open-label, phase II trial, concluded that in immunocompromised children aged ≤24 months, nirsevimab was well tolerated over 361 days and levels of antidrug antibody were low (11/100 children), with minimal effects on pharmacokinetics [31]. Fourteen children with underlying protein-losing conditions experienced a rapid decline in nirsevimab serum concentrations; however, overall nirsevimab serum exposure was consistent with previous studies in healthy children and supportive of efficacy in this population at risk of severe RSV disease (no medically attended RSV-LRTI occurred) [31].

To date, there are no published data identified for LAmAbs in other conditions associated with an increased risk for severe RSV disease in children, such as Down syndrome, cystic fibrosis, anatomic pulmonary abnormalities, or neuromuscular disorders.

### Real-world Evidence

Five prospective observational studies conducted in Spain demonstrated high rates of nirsevimab uptake ranging from 79% to 99% [28, 30, 37, 38, 40], with similarly high uptake rates reported in Luxembourg (66%–94%) [29] and Italy (65%–86%) [33]. Conversely, during the first 2023–2024 RSV season after introduction, nirsevimab uptake in the United States was low (14%) and varied across states [34, 42]. Nirsevimab has been demonstrated to be highly effective at preventing RSVH with estimates as high as 97.0% (95% CI, 87.7–99.6) in Spain (Valencia) [28], 93% (CI, 82–97) in the United States [42], and 83.0% (CI, 73.4–89.2) in France (Table 2) [39]. Moreover, effectiveness against intensive care unit (ICU) admission ranged from 85.9% to 94.4% in Spain [37, 40] and 69.6% to 75.9% in France [35, 39]. In the United States, nirsevimab was recently reported to be 89% (80%–97%) effective against medically attended RSV infection [42]. Comparison of data from the 2023–2024 season to 2018–2023 across 9 different Spanish regions revealed a significant 63.1% reduction in bronchiolitis-related hospital admissions in infants aged <6 months, which was greatest when using the extended catch-up strategy (born during the RSV season and <6 months of age at RSV season onset) versus limited catch-up (born during the RSV season and aged <3 months at RSV season onset and no catch-up strategy (birth during the RSV season) [41]. Similarly, early evidence from Luxembourg for the 2023–2024 RSV season showed a 69% decrease in RSVH in infants <6 months compared to the 2022–2023 season with a significantly reduced hospital length of stay (5.6 to 3.4 days, *P* < 0.001) [29]. No severe AEs were reported after nirsevimab administration in a real-world setting [29, 30].

**Table 2.** Uptake, Effectiveness, and Impact of Nirsevimab in Infants ≤12 Months of Age From Real-world Studies

Study	Country	Population	Sample Size	Uptake	Effectiveness	Impact	Study RoB
L**ó**pez-Lacort M et al. 2024 [28]	Spain (Valencia, Murcia and Valladolid)	All infants <9 mo eligible for nirsevimab	Nirsevimab: n = 14 106 No nirsevimab: n = 1570	79%–99% (average 90%)	RSVH: 69%–97% (pooled estimate 84.4% [95% CI, 76.8–90.0])	NR	Low
Paireau J et al. 2024 [35]	France	Healthy infants <1 mo or infants with comorbidities <5 mo at study start eligible for nirsevimab	Nirsevimab: n = 58 No nirsevimab: n = 230	20% in those with PICU admission	PICU admission: 75.9% (95% CI, 48.5–88.7)	NR	Moderate
Assad Z et al. 2024 [39]	France	All infants <12 mo eligible for nirsevimab	Nirsevimab: n = 157 No nirsevimab: n = 878	8.7% in those with RSVH	RSVH: 83% (95% CI, 73.4–89.2) PICU admission: 69.6% (42.9–83.8) Ventilatory support: 67.2% (38.6–82.5)	NR	Low
NIRSE-GAL Study [30, 32, 36, 37] 2024	Spain (Galicia)	All infants eligible for nirsevimab^a^	Nirsevimab: n = 13 320 No nirsevimab: n = 1156	96.6% in the high-risk cohort 88.5% in the catchup cohort 95.3% in the seasonal cohort^b^ (Overall: 92.0%)	RSVH: 70.7% (95% CI, 42.4–85.1) Severe RSV-LRTI with oxygen support: 80.3 (54.6–91.5) All cause bronchiolitis or bronchitis hospitalization: 46.0 (6.8–68.7) All cause LRTI hospitalization: 35.2 (−3.8–59.6)	RSVH reduced by 89.2% (IQR 89.1–91.4) in the overall cohort and by 95.2% (94.8–96.2) in the seasonal cohort (vs previous period, excluding COVID-19 period) NNT to avoid 1 RSVH: median 30 (IQR 23–30) for overall cohort and 16 (12–17) for seasonal cohort	Low
Ezpeleta G et al. 2024 [38]	Spain (Navarre)	All infants eligible for nirsevimab	Nirsevimab: n = 1083 No nirsevimab: n = 94	92%	RSVH: 88.7% (95% CI, 69.6–95.8) Accident and Emergency consultations: 87.9% (70.3–95.1) ICU: 85.9% (13.2–97.7)	NNT to avoid 1 RSVH: 15.3	Low
Barbas Del Buey JF et al. 2024 [40]	Spain (Madrid)	All infants eligible for nirsevimab	Nirsevimab: n = 29 684 No nirsevimab: n = 7383	80.08% (95% CI, 79.67–80.49)	RSVH at 30 d: 93.6% (95% CI, 89.7–96.1) RSVH at 150 d: 87.6% (67.7–95.3) ICU admission at 30 d: 94.4% (87.3–97.5) ICU admission at 90 d: 92.1% (64.0–98.3)	NNT to avoid 1 RSVH: 314.19 (95% CI, 306.22–327.99) at 30 d and 24.30 (22.31–31.61) at 150 d	Low
Consolati A et al. 2024 [33]	Italy	All infants <12 mo eligible for nirsevimab	Nirsevimab: n = 369 No nirsevimab: n = 168	65%–86% (average 69%)	RSVH risk in those not treated 8.3% (14/168) versus those treated 0% (0/369)	RVSH risk in 2023–2024 was 3.2%, versus 7% in the 2022–2023 (*P* < .001)	Low
Moline HL et al. 2024 [34, 42]	USA	Infants <8 mo at start of first RSV season	Nirsevimab: n = 136 No nirsevimab: n = 1480	14% in those with medically attended ARI	RSVH: 93% (95% CI, 82–97) Medically attended RSV ARI: 89% (79–84)	Similar to previous seasons before introduction but low uptake	Low
Ernst C et al. 2024 [29]	Luxembourg	All infants <6 m eligible for nirsevimab	Nirsevimab: n = 1277 No nirsevimab: n = 247	66%–94% (average 84%)	NR	RSVH: 69% decrease (232 in 2022/23 vs 72 in 2023/34) LOS: 39% decrease (5.6 vs 3.4 d, *P* < .001)	Low
Andina Mart**í**nez D et al. 2024 [41]	Spain (Andalusia, Aragon, Basque Country, Cantabria, Catalonia, Galicia, Madrid, Murcia, and Navarre plus the Canary Islands)	All infants <6 m eligible for nirsevimab	Nirsevimab: n = 331 No nirsevimab: n = 277	NR	NR	2018–2023 versus 2023–2024 LRTI: Overall 57.7% decrease (95% CI, 56.5–58.8; *P* < .001) Extended catch-up 61.4 (60–62.6) Limited catch- up 61.4 (60.1–62.6) No catch-up 4.8 (2.7–7.8) Acute bronchiolitis: Overall 59.2% decrease (95% CI, 57.9–60.4; *P* < .001) Extended catch-up 62.8 (61.5–64.0) Limited catch- up 34.0 (28.2–40.1) No catch-up 6.9 (4.2–10.5) Acute bronchiolitis- related hospitalization: Overall 63.1% decrease (95% CI, 60.9–65.2; *P* < .001) Extended catch-up 65.5 (63.2–67.7) Limited catch-up 46.5 (37.6–55.5) No catch-up 31.4 (20.9–43.6) Acute bronchiolitis- related PICU admissions: Overall 63.1% decrease (95% CI, 58.1–67.9; *P* < .001) Extended catch-up 66.5 (61.1–71.5) Limited catch-up 41.4 (23.5–61.1) No catch-up 40.9 (20.7–63.6)	Moderate

Abbreviations: ARI, acute respiratory illness; CI, confidence interval; ICU, intensive care unit; IQR, interquartile range; LOS, length of stay; LRTI, lower respiratory tract infection; NNT, number needed to treat; NR, not reported; PICU, pediatric intensive care unit; RoB, risk of bias; RSV, respiratory syncytial virus; RSVH, respiratory syncytial virus hospitalization.

^a^High-risk group = any child aged 6–24 mo at the start of the RSV season, with any condition placing them at high-risk for severe RSV disease; catchup group = any infant aged 0–6 mo at the start of the RSV season; Seasonal group = infants born during the RSV season.

^b^Seasonal cohort used in effectiveness analysis.

Pooled RSV surveillance data from 17 countries between 1956 and 2021 demonstrated a high degree of conservation within the nirsevimab binding site, implying that a single dose of nirsevimab can be expected to neutralize >99% of current circulating RSV strains and protect against RSV disease for 150 days postdose [46]. However, widespread use of nirsevimab may exert increased evolutionary pressure on RSV, so ongoing RSV surveillance is required to closely monitor the potential emergence of nirsevimab-neutralization escape variants [46].

### Evidence for Cost-effectiveness of LAmAbs

Nirsevimab has been found cost-effective (vs palivizumab/no prophylaxis) for use in all infants at prices ranging from USD $3.50 to $210.25 [52–64]. The lowest price (USD $3.50) at which nirsevimab was reported as cost-effective comes from a decision-support model evaluating nirsevimab in children <5 years in LMICs. This model examined different willingness-to-pay thresholds below the widely accepted cost-effectiveness threshold of 1 times the national gross domestic product *per* capita [52]. In the base case scenario (USD $3.50/dose: 77% efficacy, 5 months protection, 28%–99% coverage based on country-specific BCG vaccine uptake, and a societal perspective), the national cost per disability-adjusted life year averted for nirsevimab was <0.25 times the national gross domestic product per capita in all 133 LMICs [52 ]. Conversely, the highest price (CAD $290 [USD $210.25]) at which nirsevimab was found cost-effective was derived from a Canadian study modelling the price per dose (PPD) for nirsevimab programs in infants <12 months [61]. In the base case scenario (efficacy: medically attended RSV-LRTI 79.5%, RSVH 77.3%, ICU 86%; 5 months protection with sigmoidal decay; 100% coverage, and a societal perspective), the maximum PPD found cost effective at the willingness-to-pay threshold of <CAD $50 000 per quality-adjusted life year was CAD $290 (USD $210.25) in the birth cohort [61]. However, list prices for nirsevimab (USD $414.75 [98], CAD $952 [USD $690.20] [91] and Spain €209 [USD $236.99] [99]) exceed the nirsevimab prices found cost-effective within these modelling analyses. Furthermore, models that assumed countrywide list prices (or a similar price) for nirsevimab found all-infant programs were not cost-effective, with incremental cost-effectiveness ratios far greater than commonly used thresholds (CAD $50 000 and USD $100 000 per quality-adjusted life year) [63, 64]. However, it should be recognized that the actual purchase price of nirsevimab, as with other medications, may vary depending on negotiations with health authorities, reimbursement policies, and market conditions in each country.

Apart from country-specific differences, the wide range of prices at which nirsevimab was modelled as being cost-effective likely stems from the variability in how cost-effectiveness was derived, although the average quality score across all cost-effectiveness analyses was high (84/100) [52–64]. Numerous factors including efficacy estimates, coverage and duration of immunization (including waning), model structure, RSV seasonality, types and costs of resource use included, insufficient baseline burden of disease data in LMICs, and outcome measure all contribute to such variation, reflected by their recognition as key drivers of cost-effectiveness in the 13 models identified [52–64]. It has also been demonstrated in cost-utility models of palivizumab that long-term respiratory morbidity is a salient driver of cost-effectiveness [100, 101]. Although the effect of LAmAbs on long-term outcomes are expected to be similar to palivizumab, more confirmatory studies and data are required. Results are also inevitably affected by the model perspective and immunization program selected, as demonstrated in the aforementioned Canadian study [61]. From a healthcare perspective, as opposed to a societal perspective, the maximum cost-effective PPD fell from CAD $290 to CAD $215 in the birth cohort [61]. Conversely, maximum cost-effective PPD increased from a societal (CAD $705 and CAD $455) and healthcare perspective (CAD $615 and CAD $375) when nirsevimab was used in only higher risk infants (≤32 wGA/with CLD or CHD, and infants ≤36 wGA/with CLD or CHD, respectively) as opposed to the birth cohort [61].

### Current Guidelines for LAmAbs

Guidelines/recommendations from a limited number of countries have been published (Table 3), which pertain to LAmAb use before or during the RSV season (seasonal programs), rather than administration throughout the year. They most commonly recommend nirsevimab for all infants aged <6–8 months entering, or born during, their first RSV season, and for at-risk children aged <19–24 months entering their second RSV season, but differ regarding use in preterm healthy infants. This aligns with the World Health Organization preferred product characteristics for LAmAbs for passive immunization against RSV disease, which suggests to target all infants <6 months and encourages policy-makers to consider including high-risk children aged <24 months entering their second RSV season, based on local epidemiology and context [88].

**Table 3.** Country- and Region-specific Recommendations Regarding the Prevention of RSV Disease With Nirsevimab

Patient Group	Spain [78–80, 84, 87, 92, 93]	Luxembourg [29]	United Kingdom [96]	United States [81–83, 85, 86, 90]	Canada [91]	Latin America [95, 97]	Saudi Arabia [94]
Term infants without other comorbidities	All infants <6 mo entering, or born during, their first RSV season	All infants <6 mo entering, or born during, their first RSV season	Not recommended	All infants <8 mo entering, or born during, their first RSV season	All infants <8 m entering, or born during, their first RSV season (if cost effective—Priority 2)	All infants <6 mo entering, or born during, their first RSV season	All infants ≤12 mo entering, or born during, their first RSV season
Preterm infants without other comorbidities	Infants <12 m entering, or born during, their first RSV season born: <35 wGA, including those born <29 wGA (Spanish Society of Neonatology & Ministry of Health) Infants entering, or born during, their first RSV season born: 29–35 wGA (Spanish Society of Paediatric Infectious Disease)	No clear recommendations provided	Consider for infants during the RSV season where clinical judgment strongly indicates prophylaxis would prevent serious RSV infection	No clear recommendations provided	Infants entering, or born during, their first RSV season born <37 wGA (Priority 1)	No clear recommendations provided	All infants born: ≤29 wGA and ≤12 m of age 29–33 wGA and ≤6 mo of age 33–35 wGA infants ≤6 mo of age at the start of the RSV season or born during the RSV season with at least 1 additional risk factor
CLD/BPD	Children <24 mo at the beginning of the season in children with BPD, especially indicated in those with grades 2 and 3 and considered a priority in those who required treatment for respiratory disease in the 6 mo preceding the onset of the season	Children <24 mo with risk factors for severe RSV-LRTI	High-risk infants and young children with CLD/BPD	Children aged 8–19 mo entering their second RSV season with CLD of prematurity who required medical support (chronic corticosteroid therapy, diuretic therapy, or supplemental oxygen) any time during the 6-mo period before the start of the second RSV season	Infants born during their first RSV season or entering their first/second RSV season who have CLD, including BPD, requiring ongoing assisted ventilation, oxygen therapy or chronic medical therapy in the 6 mo prior to the start of the RSV season (Priority 1)	Children aged 8–19 mo entering their second RSV season at increased risk for severe RSV disease	Children <24 mo if still receiving medications for disease stability within 6 m from the beginning of the epidemic season
CHD	Children <24 mo at the beginning of the season in children with hemodynamically significant CHD Also, children with: Moderate or severe primary or secondary pulmonary hypertension Cardiomyopathy requiring medical treatment Severe and recurrent hemodynamically significant arrhythmia requiring treatment Children with channelopathies that carry a risk of severe arrhythmia associated with fever or infection Awaiting/received heart transplant Nonhemodynamically significant heart disease associated with other risk factors	Children <24 mo with risk factors for severe RSV-LRTI	High-risk infants and young children with hemodynamically significant CHD	Not recommended in children aged 8–19 mo entering their second RSV season with CHD	Infants born during their first RSV season or entering their first/second RSV season who have hemodynamically significant chronic cardiac disease (Priority 1)	Children aged 8–19 mo entering their second RSV season at increased risk for severe RSV disease	Children <24 mo if still receiving medications for disease stability within 6 mo from the beginning of the epidemic season
Other high-risk populations	Children <24 mo at the beginning of the season with: Severe immunosuppression Inborn errors of metabolism Neuromuscular disease Severe pulmonary malformations Genetic syndromes with significant respiratory problems Down syndrome Cystic fibrosis	Children <24 mo with risk factors for severe RSV-LRTI	High-risk infants and young children with Severe Combined Immunodeficiency Syndrome Consider for other infants during the RSV season where clinical judgement strongly indicates prophylaxis would prevent serious RSV infection	Children aged 8–19 mo entering their second RSV season with: Severe immunocompromise Cystic fibrosis patients who have either 1) manifestations of severe lung disease (previous hospitalization for pulmonary exacerbation in the first year of life or abnormalities on chest imaging that persist when stable) or 2) weight for- length that is <10th percentile American Indian and Alaska Native heritage	Infants born during their first RSV season or entering their first/second with: Cystic fibrosis with respiratory involvement and/or growth delay Severe immunodeficiency Severe congenital airway anomalies impairing clearing of respiratory secretions Neuromuscular disease impairing clearing of respiratory secretions Down syndrome (first season only) Transportation for treatment is complex, and/or whose risk of severe RSV disease intersects with established social and structural health determinants such as those experienced by some Indigenous communities across First Nations, Métis and Inuit populations (first season only) (All Priority 1)	Children aged 8–19 mo entering their second RSV season at increased risk for severe RSV disease	Consider in children <24 mo with: Anatomic pulmonary abnormalities or neuromuscular disorder with impaired ability to handle respiratory secretions Immunocompromise Down syndrome with CHD, CLD, airway clearance issues, or <35 wGA Cystic fibrosis with manifestations of severe lung disease or weight for length <10th percentile
Dosing	First RSV season: Weighing <5 kg = a 0.5 mL dose (50 mg/0.5 mL) Weighing ≥5 kg = 1 mL dose (100 mg/1 mL) Second RSV season: Weighing <10 kg = 1 mL dose (100 mg/1 mL) Weighing ≥10 kg = Single dose of 200 mg (2 × 100 mg/1 mL) using 2 different injection sites	No clear recommendations provided	Weighing <5 kg = a 0.5 mL dose (50 mg/0.5 mL) Weighing ≥5 kg = 1 mL dose (100 mg/1 mL)	First RSV season: Weighing <5 kg = a 0.5 mL dose (50 mg/0.5 mL) Weighing ≥5 kg = 1 mL dose (100 mg/1 mL) Second RSV season: Single dose of 200 mg (2 × 100 mg/1 mL) using two different injection sites	First RSV season: Weighing <5 kg = a 0.5 mL dose (50 mg/0.5 mL) Weighing ≥5 kg = 1 mL dose (100 mg/1 mL) Second RSV season: Single dose of 200 mg (2 × 100 mg/1 mL) using two different injection sites—if the child weighs <10 kg, consideration can be given to administering a single dose of 100 mg at clinical discretion	Weighing <5 kg = a 0.5 mL dose (50 mg/0.5 mL) Weighing ≥5 kg = 1 mL dose (100 mg/1 mL)	First RSV season: Weighing <5 kg = a 0.5 mL dose (50 mg/0.5 mL) Weighing ≥5 kg = 1 mL dose (100 mg/1 mL) Second RSV season: Single dose of 200 mg (2 × 100 mg/1 mL)

Abbreviations: BPD, bronchopulmonary dysplasia; CHD, congenital heart disease; CLD, chronic lung disease; LAmAbs, long-acting monoclonal antibodies; RSV, respiratory syncytial virus; wGA, weeks’ gestational age.

The US and Canadian guidelines further advise, in the context of limited supply, nirsevimab should be prioritized to protect infants and children at the highest risk for severe RSV disease (high-risk conditions, young infants <6 months) [83, 90, 91]. The US Centers for Disease Control and Prevention also recommend suspending nirsevimab use in palivizumab-eligible children aged 8–19 months, but to continue use in American Indian and Alaska Native children who are not palivizumab-eligible and who live in remote regions with known high rates of RSV among older infants and children [90].

Furthermore, the American Academy of Pediatrics offers guidance on dosing nirsevimab in relation to palivizumab [83]. If palivizumab was administered initially for the season and <5 doses were administered, the infant should receive 1 dose of nirsevimab (no minimum interval between palivizumab and nirsevimab doses) and no further palivizumab. If palivizumab was administered in season 1 and the child is eligible for RSV prophylaxis in season 2, the child should receive nirsevimab in season 2, if available [83].

## CONSENSUS RECOMMENDATIONS FOR RSV DISEASE PROPHYLAXIS WITH LAMABS

Considering all available evidence and the existing guidelines, the ARMADA Taskforce have developed the following recommendations for use of LAmAbs for the prevention of RSV disease in young children (Table 4). These recommendations also apply if the mother did not receive RSV vaccine during pregnancy, her vaccination status is unknown, or the infant was born within 14 days of maternal RSV vaccination. A LAmAb can be administered concurrently with other childhood immunizations including BCG and hepatitis B.

**Table 4.** Summary of ARMADA Taskforce Recommendations

Recommendation	Level of Evidence^a^	Strength of Recommendation (GRADE^b^)	Consensus
Term infants without other comorbidities Nirsevimab is recommended for: All infants <8 mo of age at the start of, or born during, their first RSV season	1a	A	87.5% (Fully agree: 68.75% Partially agree: 18.75%)
Preterm infants without other comorbidities Nirsevimab is recommended for infants: <37 wGA and <12 mo at the start of, or born during, their first RSV season	1a	A	100% (Fully agree: 100%)
Children with CLD/BPD Nirsevimab is recommended for: Children <24 mo entering their second season with any grade of CLD/BPD	1b	B	93.75% (Fully agree: 81.25% Partially agree: 12.50%)
Children with HS-CHD Nirsevimab is recommended for: Children <24 mo entering their second season with uncorrected, palliated cyanotic or acyanotic HS-CHD associated with documented moderate or severe pulmonary hypertension, and/or a requirement for daily medication to manage congestive heart failure or failure to thrive based on CHD status	1b	B	100% (Fully agree: 93.75% Partially agree: 6.25%)
Children with other high-risk conditions Nirsevimab is recommended for children <24 mo entering their second season who have increased risk for severe RSV. These include: Severe immunosuppression Inborn errors of metabolism Neuromuscular disease Severe pulmonary malformations Genetic syndromes with significant respiratory problems Down syndrome Cystic fibrosis American Indian and Alaska Native heritage; Māori and Pacific ethnicities	5^c^	D^c^	93.75% (Fully agree: 87.5% Partially agree: 6.25%)
Dosing (nirsevimab) Weighing <5 kg = a 0.5 mL dose (50 mg/0.5 mL) given intramuscularly Weighing ≥5 kg = 1 mL dose (100 mg/1 mL) given intramuscularly Single dose of 200 mg (2 × 100 mg/1 mL) using 2 different injection sites during the second season only given intramuscularly Administer soon after birth for infants born during the RSV season, or just prior to the RSV season onset for infants born outside the season In endemic countries a decision should be made locally as to whether to administer prophylaxis throughout the year or to coincide with annual peak RSV incidences	1a	A	100% (Fully agree: 93.75% Partially agree: 6.25%)

Abbreviations: BPD, bronchopulmonary dysplasia; HS-CHD, hemodynamically significant congenital heart disease; CLD, chronic lung disease; RCT, randomized controlled trial; RSV, respiratory syncytial virus; wGA, weeks’ gestational age.

^a^1a: systematic review of RCTs; 1b: individual RCT; 2a: systematic review of cohort studies; 2b: individual cohort study; 2c: outcomes research/registries; 3a: systematic review of case-control studies; 3b: individual case-control study; 4: case series; 5: expert opinion.

^b^GRADE—A: consistent with level 1 studies (high quality); B: consistent with level 2 or 3 studies or extrapolations from level 1 studies (moderate quality); C: level 4 studies or extrapolations from level 2 or 3 studies (low quality); D: level 5 evidence (very low quality).

^c^Supported by 2B to 4C evidence for palivizumab [4].

## DIRECTIONS FOR FUTURE RESEARCH

There are 5 key areas of research that should be prioritized with respect to LAmAbs:

1. The effectiveness of LAmAbs in reducing severe RSV-LRTI in children with underlying medical conditions (particularly CLD/BPD, CHD, Down syndrome, cystic fibrosis, anatomic pulmonary abnormalities, neuromuscular disorders, and immunocompromise) and for the same groups of children entering their second RSV season
2. The impact of LAmAbs on long-term respiratory morbidity including recurrent LRTI, wheezing, asthma, and lung function impairment
3. The use of LAmAbs in programs in which maternal RSV vaccine is also deployed. Of note, maternal RSV vaccine uptake rates between 17.8% and 62.5% have recently been reported across Argentina, the United Kingdom, Uruguay, and the United States [102, 103] and data on the combined use of maternal RSVpreF vaccine and nirsevimab are rapidly emerging [104–107].
4. Postimplementation surveillance for RSV disease through 2 years of age and for possible RSV escape mutants globally
5. Impact on non-RSV outcomes such as all cause LRTI, otitis media, and antibiotic prescription

## DISCUSSION AND CONCLUSIONS

These up-to-date consensus recommendations have been developed by the ARMADA Taskforce predicated on a systematic evaluation of the existing evidence, current national guidelines, and expert experiences in the management of RSV-LRTI in infants and children. The recommendations broadly align with the existing LAmAb guidelines used in countries such as the United States, Spain, and Canada and are supported by a growing evidence base, including RCTs, real-world evidence, and pooled analyses. Importantly, LAmAbs have appeared cost-effective across HICs and LMICs, albeit at a wide range of prices. These prices included significantly lower prices than those at which LAmAbs are likely to be available, especially in LMICs, and thus the cost of LAmAb is a factor that must be considered by individual countries when adopting nirsevimab. Affordable access to LAmAbs is more challenging in LMICs and successful implementation will require strong collaboration between stakeholders, including pharmaceutical companies, distributors, funders, and public health programs [108].

The success of LAmAbs is likely to be impacted by the availability and use of the maternal RSV prefusion F protein-based (RSVpreF) vaccine, particularly in terms of vaccination acceptance and coverage. Administration of both products is unnecessary for most infants. Nirsevimab may be indicated despite maternal vaccination under special circumstances, but recommendations in this regard vary across countries. Such circumstances include, but are not limited to, those deemed at substantially increased risk for severe RSV-LRTI, those in whom transplacental transfer of antibody may be suboptimal (eg, if birth occurred <2 weeks after antenatal administration of RSVpreF or in mothers’ with uncontrolled HIV or malaria), or if a second pregnancy follows a first pregnancy in which the mother received RSV immunization [83, 86, 96, 109]. The advantages and disadvantages of maternal RSVpreF vaccination and LAmAbs may be considered alongside patient preference to determine the optimal immunization strategy [86, 110].

The ARMADA Taskforce strongly endorse the implementation of LAmAb programs to prevent RSV-LRTI in infants and young children. These evidence-based recommendations can be used as a universal template to inform the development of regional and national society guidelines worldwide. Widespread global use of LAmAb is essential to protect all infants and children against RSV disease. Product access and affordability in LMICs is crucial to reduce global inequity and the global burden of severe RSV disease and mortality.

## Supplementary Material

ofaf396_Supplementary_Data

## Supplementary Data

Supplementary materials are available at *Open Forum Infectious Diseases* online. Consisting of data provided by the authors to benefit the reader, the posted materials are not copyedited and are the sole responsibility of the authors, so questions or comments should be addressed to the corresponding author.

## Notes

***Acknowledgments.*** All authors contributed to the development of the publication and maintained control over the final content.

***Financial support.*** Financial support for this study was provided by Sanofi and AstraZeneca. The sponsors had no role in the selection of ARMADA participants, the preparation of the manuscript, or the decision to submit for publication.
