# Supplementary material for: Systematic Review and Expert Consensus on the Use of Long-acting Monoclonal Antibodies for Prevention of Respiratory Syncytial Virus Disease: ARMADA (Advancing RSV Management And Disease Awareness) Taskforce
Source: Open Forum Infect Dis. 2025 Jul 2;12(7):ofaf396. doi: 10.1093/ofid/ofaf396 (PMC12290402; doi:10.1093/ofid/ofaf396)
Supplement: ofaf396_Supplementary_Data [file ofaf396_supplementary_data.pdf]

Supplementary Appendix

ARMADA (Advancing RSV Management And Disease Awareness) Taskforce: Systematic review and expert consensus on the use of long-acting monoclonal antibodies for prevention of respiratory syncytial virus disease

Paolo Manzoni, Eugenio Baraldi, Fabio Midulla, Olivier Claris, Sandro Dessardo, Terho Heikkinen, Richard Thwaites, Bosco Paes, Xavier Carbonell-Estrany, Dmytro Dobryanskyy, Merih Cetinkaya, Adel S. Al Harbi, Ji-Man Kang, Anne Goh Eng Neo, Hsin Chi, Guilherme Sant'Anna, Mónica Villa Guillén, Gonzalo Luis Mariani, Marco Aurelio Palazzi Safadi, Soledad Urzua, Heather J Zar, Pierre Goussard, Barry Rodgers-Gray, Nicola Waghorne, Manuel Sanchez Luna

Contents

Supplementary File 1: ARMADA: An international expert consensus on long-acting monoclonal antibodies for respiratory syncytial virus prevention - Systematic Literature Review Protocol 2

Supplementary File 2: Characteristics of Included Studies 7

Supplementary File 3: Risk of Bias Assessments 18

References 21

Supplementary File 1: ARMADA: An international expert consensus on long-acting monoclonal antibodies for respiratory syncytial virus prevention - Systematic Literature Review Protocol

1. Background

Respiratory syncytial virus (RSV) is the leading viral cause of childhood pneumonia and is estimated to cause approximately 12.9 million lower respiratory tract infections resulting in 2.2 million hospitalisations and >66,000 deaths annually in children <1 year worldwide.[1,2] The introduction of long-acting monoclonal antibodies for RSV prevention has the potential to have a dramatic effect on reducing the intensity of RSV epidemic waves and concomitant capacity surges on paediatric acute care systems and thereby profoundly impact the global burden of RSV. To maximise the benefits of long-acting monoclonals it is essential that their deployment is guided by clear and evidence-based recommendations.

A panel of international RSV experts has been formed with the aim of developing an expert- and evidence-driven consensus on long-acting monoclonal antibodies for RSV prevention.

This protocol addresses how a systematic literature review (SLR) will be conducted to identify published evidence on long-acting monoclonal antibodies for RSV prevention. The SLR will use systematic and explicit methods to identify, select, and critically appraise relevant research, and to collect, analyse and report data from the studies that are included in the review according to the PRISMA guidelines.[3]

2. Review Question

The SLR will address the following question:

What is the evidence on long-acting monoclonal antibodies for RSV prevention?

3. Databases

The following electronic databases will be searched:

- PubMed (Medline)
- Embase
- The Cochrane Library

In addition, other relevant studies and evidence will be identified *via* bibliographies/reference lists of key articles and targeted web searches for non-indexed articles, theses and dissertations, meeting abstracts, letters, research and committee reports, and government reports *etc* (the 'grey literature').[4]

No time limits will be set on the database/grey literature searches. No language limits will be set on database searches/grey literature, with the caveat that English translations of at least the abstract must be available.

3. Search terms

Search terms (combined with 'Medical Subject Headings' [MeSH] in PubMed and 'Emtree Subject Headings' in Embase) will include:

Concept	Key word(s)	Search Terms (all fields unless stated)	PubMed Results*	Embase	Cochrane Library
**Disease**	1. RSV	RSV OR "respiratory syncytial virus" OR "lower respiratory tract infection" OR bronchiolitis OR "acute respiratory tract infection"	51,672	110,551	5,521

		OR LRTI OR LRI OR ARTI OR ARI<br><br>OR MeSH: "Respiratory Syncytial Virus"/ Emtree: "Respiratory Syncytial Pneumovirus"			
**Intervention**	2. Long-acting monoclonal antibodies	immunoprophylax* OR prophylax* OR monoclonal antibod* OR nirsevimab OR Beyfortus OR MK-1654 OR clesrovimab OR motavizumab OR suptavumab OR MEDI8897	478,763	659,160	54,019
	3.	1 AND 2	3,036	6,250	**383**
**Filters**	4. human, child (birth to 18 years)		**1,426**	1,471 **(546 with Medline records removed)**	N/A

*Searches undertaken on 20 February 2024

4. Inclusion and Exclusion Criteria

The conditions/domain being reviewed is RSV infection. The PICOS (Population, Intervention, Comparison, Outcomes and Study Design) tool will be used to identify relevant references to the systematic review outcome using the following inclusion and exclusion criteria:

PICOS	Inclusion criteria	Exclusion criteria
**Population**	- Children (<5 years) requiring RSV prophylaxis	- Non-human<br>- Children (>5 years) and adults
**Intervention**	- Long-acting monoclonal antibodies for RSV prevention (*e.g.* nirsevimab)	- Other RSV prophylactic drugs<br>- Supportive RSV treatments
**Comparator**	- Placebo or any other RSV prophylaxis (*e.g.* palivizumab)<br>- Historical controls	- No comparator group
**Outcomes**	Primary:<br>- Medically-attended RSV infections<br>Secondary:<br>- Burden/morbidity (*e.g.* hospitalisation, ICU admission, long-term sequelae *etc.*)<br>- Mortality<br>- Safety<br>- Cost-effectiveness<br>- Immunogenicity (*e.g.* measurement of antibody levels [anti-RSV neutralising antibodies] and their persistence over time; assessment of the potential impact of long-acting monoclonal antibodies on the development of the child's natural immune response to RSV)<br>- Elicitation of anti-drug antibodies (ADA)	- Any data not related to RSV or long-acting monoclonal antibodies for RSV prevention
**Study Design**	- Any relevant study design (*e.g.* randomised controlled trials, cohort studies, guidelines)	- Preclinical studies, case studies, protocols, non-systematic reviews

5. Data extraction

After removal of duplicates, studies will be selected for inclusion in the review using a two-phase approach:

Phase 1 - the abstracts of potentially relevant citations identified from the electronic searches will be assessed separately by two experienced reviewers to confirm relevance and inclusion in the study according to the inclusion criteria

Phase 2 - the full texts over those citations identified as relevant in phase 1 will be assessed separately by two experienced reviewers to confirm relevance and inclusion in the review according to the inclusion criteria. If a consensus cannot be reached on a citation, a third senior researcher will make the decision.

A PRISMA diagram will be used to summarise the exclusions and the reasons for exclusion during phases 1 and 2. An Excel spreadsheet will also provide a reference list of all citations, detailing exclusions and the reasons for these exclusions.

Data will be extracted from the full-text of all relevant articles identified in Phase 2 by one reviewer, and quality checked by a second reviewer. All information will be inserted into an agreed Excel data extraction table template. Corresponding authors of any papers with data that require clarification will be contacted.

The data extraction table will record the following information: citation; study aim; study design; methods; results; conclusion; study quality score.

6. Quality assessment

The risk of bias and confounding will be assessed for any relevant randomised clinical trials using the Cochrane Collaboration risk of bias tool.[5] For observational studies using the RTI Item Bank[6] will be applied. For cost-effectiveness analyses, the Quality of Health Economic Studies List will be used.[7]

7. Strategy for data synthesis

Relevant information from the SLR data extraction table will be used to provide narrative synthesis of the evidence on long-acting monoclonal antibodies for RSV prevention. Along with a PRISMA flow diagram of search results, this summary will be included in expert- and evidence-driven consensus publication and ultimately help form the overall recommendations. The strengths and weaknesses of the evidence will be evaluated, and considered when rating the strength of evidence for each recommendation. Potential areas for future research will also be identified.

8. Analysis of subgroups or subsets

Depending on the evidence identified, analysis may be split into different patient groups such as preterm infants without comorbidities, those with chronic lung disease, those with congenital heart disease and other high-risk populations. Depending on the evidence collated, analysis based on different age groups may also be performed.

9. Dissemination plans

Evidence from the SLR will be used to help develop an expert- and evidence-driven consensus on long-acting monoclonal antibodies for RSV prevention for publication in a leading paediatric and/or infectious disease journal. In addition to publishing the consensus in a leading journal, to maximise impact and dissemination, the plan would be to present the recommendations at an international conference. It is envisaged that these recommendations would provide a template or blueprint to inform the development of regional and national society guidelines across the world.

10. Review team

- Prof Paolo Manzoni
  - Department of Public Health and Pediatric Sciences, University of Torino School of Medicine, Turin, Piedmont, Italy
  - Division of Paediatrics and Neonatology, Degli Infermi Hospital, Ponderano, Italy
- Prof Eugenio Baraldi
  - Department of Women's and Children's Health, University Hospital of Padova, Veneto, Italy
  - Institute of Pediatric Research, "Città della Speranza", Padova, Veneto, Italy
- Prof Fabio Midulla
  - Department of Pediatrics and Pediatric Neuropsychiatry. Sapienza University of Rome, Rome, Italy
- Prof Manuel Sanchez Luna
  - Neonatology Division, University General Hospital Gregorio Maranon, Complutense University of Madrid, Madrid, Spain
- Prof Olivier Claris
  - Hospices Civils de Lyon, Hôpital Femme Mère Enfant, Service de Réanimation Néonatale, Bron, France
  - EA 4129, Université Claude Bernard Lyon 1, Lyon, France
- Prof Sandro Dessardo
  - Department of Pediatrics, University Hospital Centre Zagreb, Croatia
- Prof Terho Heikkinen
  - Department of Pediatrics, University of Turku and Turku University Hospital, Turku, Finland
- Dr Richard Thwaites
  - The Neonatal Unit, Royal Stoke University Hospitals, Stoke-on-Trent, UK
- Prof Dmytro Dobryanskyy
  - Department of Pediatrics, Lviv National Medical University, Lviv, Ukraine
- Prof Merih Cetinkaya
  - Department of Neonatology, Health Sciences University, Basaksehir Cam and Sakura City Hospital, Istanbul, Turkey
- Prof Adel S. Al Harbi
  - Department of Pediatrics, Prince Sultan Military Medical City, Alfaisal University, Riyadh, Saudi Arabia
- Prof Ji-Man Kang
  - Department of Pediatrics, Severance Children's Hospital, Yonsei University College of Medicine, Seoul, South Korea
  - Institute for Immunology and Immunological Diseases, Yonsei University College of Medicine, Seoul, South Korea
- Prof Anne Goh Eng Neo
  - Department of Pediatrics, KK Women's and Children's Hospital, Singapore
- Prof Hsin Chi
  - Department of Pediatrics, MacKay Children's Hospital, Taipei, Taiwan
  - MacKay Memorial Hospital, Taipei, Taiwan
- Prof Guilherme Sant'Anna
  - Department of Pediatrics, McGill University Health Centre, Montreal, Canada
- Dr Mónica Villa Guillén
  - National Institute of Health Children´s Hospital of Mexico Federico Gómez, Mexico City, Mexico
  - President of the National Federation of Neonatology of Mexico
- Prof Gonzalo Luis Mariani
  - Division of Neonatology, Departments of Pediatrics, Instituto Universitario Hospital Italiano, Buenos Aires, Argentina
- Prof Marco Aurelio Palazzi Safadi

    - Department of Pediatrics, Santa Casa de São Paulo School of Medical Sciences, Brazil
- Dr Soledad Urzua
    - Department of Neonatology, School of Medicine, Pontificia Universidad Catolica de Chile, Santiago, Chile
- Prof Heather Zar
    - Department of Paediatrics and Child Health, Red Cross War Memorial Children's Hospital, Cape Town, South Africa
    - SA Medical Research Council Unit on Child and Adolescent Health, University of Cape Town, Cape Town, South Africa
- Prof Pierre Goussard
    - Department of Paediatrics and Child Heath at Stellenbosch University, Stellenbosch, South Africa
    - Paediatric Pulmonology and Paediatric Intensive Care at Tygerberg Hospital, Parow, South Africa

Dr Barry Rodgers-Gray, Violicom Medical Limited, Aldermaston, UK – Reviewer 1
Dr Nicola Waghorne, Violicom Medical Limited, Aldermaston, UK – Reviewer 2

11. Organisational affiliation of the review

Violicom Medical Limited

12. Funding sources/sponsors

Sanofi

13. Conflicts of interest

PM has received research funding and/or compensation as advisor/lecturer from AstraZeneca, Moderna, GSK, and Sanofi and is a member of RESVINET. EB has received fees for lectures and advisory boards from Sanofi, AstraZeneca, and Chiesi. MSL has received consultancy fees, and paid lectures from AstraZeneca and Sanofi, and paid lectures from Pfizer. TH has received fees for advisory boards and lectures from Sanofi, MS Pharma and Pfizer, and for an independent data monitoring committee for Moderna. RT has completed consultancy work for AstraZeneca. AGEN has received honoraria for participating in advisory boards from Sanofi and AstraZeneca. MC has been an advisory board member for AstraZeneca. MAPS has received honoraria for lectures and/or participation in advisory boards or data monitoring committees from Sanofi, AstraZeneca, Abbvie, Pfizer and GSK. BRG/NW's employer have previously received payment for work on various projects from AbbVie, AstraZeneca, and Sanofi. FM, OC, SD, DD, ASAH, JMK, HC, GS, MVG, GLM, SU, HZ and PG have nothing to declare.

14. Anticipated start date

February 2024

15. Anticipated completion date

March 2024

Supplementary File 2: Characteristics of Included Studies

Study	Design	Objectives	Country	Population	LAmAb	Comparator	Outcomes of interest
**Drysdale SB et al. 2023**[8]	RCT	To determine the efficacy and safety of a single intramuscular injection of nirsevimab as compared with standard care in preventing RSVH in infants ≤12 months of age who are not eligible to receive palivizumab.	France, Germany, and the United Kingdom	Healthy infants ≤12 months of age born at ≥29 wGA	Nirsevimab (n=4037)	SoC/No intervention (n=4021)	RSVH, very severe RSV-LRTI, hospitalisation for LRTI from any cause, and AEs
**Ahani B et al. 2023**[9]	RCT	To genotypically evaluate RSV infections occurring in two pivotal nirsevimab clinical trials (Phase 2b: NCT02878330; MELODY: NCT03979313), characterise substitutions in the nirsevimab binding site, and determine their effects on nirsevimab.	21-23 countries across northern and southern hemispheres	Healthy infants ≤12 months of age born between 29 wGA and full term	Nirsevimab (n=100)	Placebo (n=142)	Genotypic and phenotypic resistance / nirsevimab binding site substitutions
**Domachowske JB et al. 2023**[10]	RCT	To report the safety and PK extrapolation of efficacy among children with CHD or CLD following administration of a second nirsevimab dose prior to their second RSV season.	25 countries across northern and southern hemispheres	Infants who had uncorrected, partially corrected, or medically treated CHD or CLD warranting therapeutic intervention within 6 months	Nirsevimab both season (n=180)	Palivizumab then nirsevimab (n=40)<br><br>Palivizumab both seasons (n=42)	AEs, PKs and ADAs
**Wilkins D et al. 2023**[11]	RCT	To characterise baseline maternal RSV antibody levels in preterm and full-term infants entering their first RSV season; (2) determine the level and duration of RSV NAb levels provided by nirsevimab; (3) investigate the incidence of clinical (symptomatic) and subclinical (asymptomatic) RSV infections in the first year of life; and (4) evaluate whether infants can mount a natural immune response against RSV in the presence of nirsevimab.	21-23 countries across northern and southern hemispheres	Healthy infants ≤12 months of age born between 29 wGA and full term	Nirsevimab (n=1427)	Placebo (n=716)	Level and duration of RSV NAb levels
**Muller WJ et al. 2023**[12]	RCT	To evaluate the efficacy and safety of nirsevimab in healthy late-preterm and term infants entering their first RSV season using the full enrolment cohort in the MELODY trial.	31 countries across northern and southern hemispheres	Healthy infants ≤12 months of age born at ≥$35^{0/7}$ wGA	Nirsevimab (n=1998)	Placebo (n=960)	Medically-attended RSV- LRTI, RSVH, very severe medically-attended RSV-LRTI, medically-attended LRTI of any cause, and hospitalisation for LRTI of any cause, and AEs

**Domachowske J et al. 2022[13]**	RCT	To report the safety and pharmacokinetics of nirsevimab through the first RSV season in a study spanning two RSV seasons.	25 countries across northern and southern hemispheres	Preterm cohort: Infants eligible for palivizumab, born ≤35 wGA, and who did not have CHD or CLD of prematurity<br>CHD–CLD cohort: Infants who had uncorrected, partially corrected, or medically treated CHD or CLD warranting therapeutic intervention within 6 months	Nirsevimab (n=616)<br><br>(Preterm: n=407 & CHD-CLD: n=209)	PVZ (n=309)<br><br>(Preterm: n=20 & CHD-CLD: n=101)	AEs, PKs and ADAs
**Hammitt LL et al. 2022[14]**	RCT	To evaluate the efficacy and safety of nirsevimab in healthy late-preterm and term infants entering their first RSV season.	21 countries across northern and southern hemispheres	Healthy infants ≤12 months of age born at $\geq 35^{0/7}$ wGA	Nirsevimab (n=994)	Placebo (n=496)	Medically-attended RSV-LRTI, RSVH, , medically-attended LRTI of any cause, hospitalisation for LRTI of any cause AEs, PKs, and ADAs.
**Griffin MP et al. 2020[15]**	RCT	To evaluate a single dose of nirsevimab prophylaxis in healthy preterm infants entering their first RSV season.	23 countries across northern and southern hemispheres	Healthy infants ≤12 months of age born at $29^{0/7}$ - $34^{6/7}$ wGA	Nirsevimab (n=969)	Placebo (n=484)	Medically-attended RSV-LRTI, RSVH, , medically-attended LRTI of any cause, hospitalisation for LRTI of any cause AEs, PKs, ADAs, nirsevimab resistance.
**Domachowske JB et al. 2018[16]**	RCT	To evaluate the safety and PK of MEDI8897 when administered to healthy preterm infants as a single 10, 25 or 50 mg IM dose.	USA, South Africa and Chile	Healthy infants ≤12 months of age born at $32^{0/7}$ - $34^{6/7}$ wGA	Nirsevimab (n=71)<br><br>(10 mg n=8, 25 mg n=31, 50 mg n=32)	Placebo (n=18)	Serum concentrations, RSV-NAb levels, AEs
**Madhi 2024[17]**	RCT	To evaluate the safety, tolerability, pharmacokinetics, and incidence of ADAs of single ascending doses of clesrovimab in healthy pre-term and full-term infants.	6 countries across northern and southern hemispheres	Healthy infants ≤8 months of age born between 29 wGA and full term	Clesrovimab (n= 145)<br><br>(20 mg n=8, 50 mg n=31, 75 mg n=41, 100 mg in preterm n=32, 100 mg in full term n=33)	Placebo (n=38)	AEs, serum concentrations, ADA
**Dagan R et al. 2022[18]**	RCT	To evaluate the theoretical risk of antibody-dependent enhancement in the setting of low nirsevimab concentrations and to report the incidence and disease severity of medically-attended RSV-LRTI during the second season.	21 countries across northern and southern hemispheres	Healthy infants ≤12 months of age born at $\geq 35^{0/7}$ wGA	Nirsevimab (n=964)	Placebo (n=482)	Medically-attended RSV-LRTI, RSVH, medically-attended LRTI of any cause, and hospitalisation for LRTI of any cause

**Zar HJ et al. 2024**[19]	RCT	To evaluate the safety, tolerability and efficacy of clesrovimab compared to placebo	22 countries across northern and southern hemispheres	Healthy preterm and full term (≥29 wGA)	Clesrovimab (n=2,411)	Placebo (n-1,203)	AEs, Medically-attended RSV-LRTI, RSVH
**Zar HJ et al. 2024**[20]	RCT	To evaluate the safety, tolerability and efficacy of clesrovimab compared to palivizumab	27 countries across northern and southern hemispheres	Infants ≤12 months and recommended to receive palivizumab (≤35 wGA, CLD, HS-CHD)	Clesrovimab (n=450)	Palivizumab (n=451)	AEs, Medically-attended RSV-LRTI, RSVH, PKs
**Arbetter D et al. 2024**[21]	RCT	This post-hoc exploratory analysis of infants entering their first RSV season in the MELODY trial examined the incidence of LRTI from RSV and other respiratory pathogens.	31 countries across northern and southern hemispheres	Healthy infants ≤12 months of age born at $\geq 35^{0/7}$ wGA	Nirsevimab (n=337)	Placebo (n=224)	Medically-attended RSV-LRTI, Medically-attended non-RSV-LRTI
**Domachowske JB et al. 2023**[22]	Uncontrolled single-dose study	To evaluate the safety, PK, and emergence of ADAs in immunocompromised children ≤24 months of age.	8 countries across northern and southern hemispheres	Immunocompromised children ≤24 months of age	Nirsevimab (n=100)	N/A	AEs, ADAs, PKs, and medically-attended RSV-LRTI
**López-Lacort M et al. 2024**[23]	Observational study	To provide the early estimates of the effectiveness of nirsevimab against hospital admission for RSV-LRTI in infants (<9 months old) in three autonomous regions of Spain.	Spain	All infants <9 months of age eligible for nirsevimab	Nirsevimab (n=14,106)	No nirsevimab (n=1570)	RSVH, uptake
**Ernst C et al. 2024**[24]	Observational study	To estimate the neonate coverage of nirsevimab immunisation in Luxembourg up to mid-December 2023 and to investigate the effect of this immunisation on children under 5 years of age, by comparing RSV-related paediatric hospitalisation data between weeks 39–52 of 2022 (pre-immunisation period) and of 2023 (post-immunisation) respectively, at Luxembourg's national paediatric hospital.	Luxembourg	Children < 5 years of age (focus on < 6 months)	Nirsevimab (n=1277)	No nirsevimab (n=247)	RSVH, uptake, AEs, recommendations for use
**Martinón-Torres F et al. 2023**[25]	Observational study	To share our experiences of integrating nirsevimab into the regional immunisation programme in Galicia, Spain.	Spain	All infants eligible for nirsevimab	Nirsevimab (n=7,241)	No nirsevimab (n=1,426)	Uptake and AEs
**Consolati A et al. 2024**[26]	Observational study	To describe the effectiveness and safety of the universal prevention program for RSV bronchiolitis using the mAb nirsevimab in children resident in Valle d'Aosta born during the 2023‑2024 epidemic season.	Italy	All infants <12 months of age eligible for nirsevimab	Nirsevimab (n=369)	No nirsevimab (n=168)	RSVH, uptake, AEs

**Moline HL et al. 2024**[27]	Observational study	To provide the first USA estimate for post-introduction nirsevimab effectiveness among infants during their first RSV season.	USA	Infants <8 months at start of first RSV season eligible for nirsevimab	Nirsevimab (n=59)	No nirsevimab (n=640)	RSVH , uptake
**Paireau J et al. 2024**[28]	Observational study	To estimate nirsevimab effectiveness against severe cases of RSV bronchiolitis hospitalised in PICU in metropolitan France from 15 September 2023 to 31 January 2024.	France	Healthy infants <1 month or infants with comorbidities <5 months at study start eligible for nirsevimab	Nirsevimab (n=58)	No nirsevimab (n=230)	RSV PICU admission, uptake
**Ares-Gómez S et al. 2024**[29]	Observational study	To report the outcomes of nirsevimab use up to 3 months after its practical implementation.	Spain	All infants eligible for nirsevimab	Nirsevimab (n=9408)	No nirsevimab (n=851)	RSVH, severe RSV-LRTI with oxygen support/ICU admission/mechanical ventilation, all-cause LRTI hospitalisation, all-cause hospitalisation, uptake, AEs
**Mallaha N et al. 2024**[30]	Observational study	To provide full-season analysis of nirsevimab effectiveness and impact using data collected between Sept 25, 2023, and April 15, 2024, from infants born up to March 31, 2024.	Spain	All infants eligible for nirsevimab	Nirsevimab (n=13,320)	No nirsevimab (n=1,156)	RSVH, severe RSV-LRTI with oxygen support, all-cause bronchiolitis or bronchitis hospitalisation, all-cause pneumonia hospitalisation, all-cause LRTI hospitalisation, all-cause hospitalisation, uptake, AEs
**Ezpeleta G et al. 2024**[31]	Observational study	To evaluate the effectiveness of nirsevimab in preventing RSVH and the impact of a strategy of immunisation at birth.	Spain	All infants eligible for nirsevimab	Nirsevimab (n=1083)	No nirsevimab (n=94)	RSVH, ICU admission, A&E consultation, uptake, AEs
**Assad Z et al. 2024**[32]	Observational study	To assess the real-world effectiveness of nirsevimab therapy against RSVH among infants <12 months of age.	France	All infants <12 months of age eligible for nirsevimab	Nirsevimab (n=157)	No nirsevimab (n=878)	RSVH, ICU admission, mechanical ventilation, uptake
**Barbas Del Buey JF et al. 2024**[33]	Observational study	To estimate the effectiveness of nirsevimab over time for the prevention of respiratory episodes treated at different levels of care.	Spain	All infants eligible for nirsevimab	Nirsevimab (n=29,684)	No nirsevimab (n=7,383)	RSVH, ICU admission, A&E consultation, primary care consultation, uptake
**Andina Martínez D et al. 2024**[34]	Observational study	To assess how RSV prophylaxis affected the number of infants presenting to paediatric emergency departments with acute respiratory infections.	Spain	All infants <6 months of age eligible for nirsevimab	Nirsevimab (n=331)	No nirsevimab (n=277)	RSVH, LRTI, bronchiolitis, bronchiolitis-related hospitalisation, bronchiolitis-related PICU, uptake

**Moline HL et al. 2024**[35]	Observational study	To compare the epidemiology and disease burden of medically attended RSV-associated acute respiratory illness (ARI) among children younger than 5 years during the 2023-2024 RSV season with 3 prepandemic RSV seasons (2017-2020), estimate nirsevimab effectiveness against medically attended RSV-associated ARI, and compare nirsevimab binding site mutations among circulating RSV in infants with and without nirsevimab receipt.	USA	Children <5 years of age with medically-attended RSV-ARI (<8 months for nirsevimab effectiveness analysis)	Nirsevimab (n=402, 1st season)<br><br>For effectiveness analysis: nirsevimab (n=136)	No nirsevimab (n=2,587, 1st season)<br><br>For effectiveness analysis: No nirsevimab (n=1,480)	Medically-attended ARI, RSVH, uptake
**Dirección Xeral de Saúde Pública. 2024**[36]	Observational study/public health report	To report on nirsevimab immunisation coverage and compare the number and rates of hospitalisations in the current RSV season 2023-2024 with those of previous seasons since 2017.	Spain	All infants eligible for nirsevimab	Nirsevimab (n=12,472)	No nirsevimab (n=1,337)	RSVH, uptake, AEs
**Wilkins D et al. 2023**[37]	Pooled analysis	To examine prospective RSV surveillance data to assess the geotemporal prevalence of RSV A and B, and functionally characterise the effect of the nirsevimab binding-site substitutions identified between 2015 and 2021.	17 countries across northern and southern hemispheres	NR	3 prospective observational studies and NCBI GenBank<br><br>Prospective (n=5675) and retrospective (n=3626) RSV fusion-protein sequences	N/A	Nirsevimab binding-site conservation
**Sun M et al. 2023**[38]	Pooled analysis	To compare the efficacy and safety of mAbs for the prevention of RSV infection in infants and children.	32 countries across northern and southern hemispheres	Healthy term and preterm infants, and infants < 24 months with CLD or CHD	14 RCTs<br><br>Nirsevimab, motavizumab, palivizumab, suptavumab and placebo involving a total of 18,042 participants		RSVH, RSV-related infection, all-cause mortality, AEs, ICU admission, supplemental oxygen use, and mechanical ventilation use.
**Simões EAF et al. 2023**[39]	Pooled analysis	To assess the efficacy of nirsevimab using a weight-banded dosing regimen in infants born between 29 weeks gestational age and full term.	21-23 countries across northern and southern hemispheres	Healthy infants ≤12 months of age born between 29 wGA and full term (extrapolated efficacy in infants with CLD, CHD, or extreme preterm birth [<29 wGA])	2 RCTs<br><br>Nirsevimab (n=1546)	Placebo (n=786)	Medically-attended RSV- LRTI, RSVH, very severe medically-attended RSV-LRTI, medically-attended LRTI of any cause, and hospitalisation for LRTI of any cause, PKs and phenotypic/genotypic analysis
**Abram ME et al. 2022**[40]	Pooled analysis	To summarise resistance analyses of all RT-PCR-confirmed RSV isolates from healthy term and preterm infants through 150 days post dose.	21-23 countries across northern and southern hemispheres	Healthy infants ≤12 months of age born between 29 wGA and full term	2 RCTs<br><br>Nirsevimab (n=19)	Placebo (n=51)	Nirsevimab resistance / binding-site substitutions

**Turalde-Mapili MWR et al. 2023**[41]	Pooled analysis	To determine the efficacy and safety of nirsevimab in preventing RSV infection among infants using a review of relevant clinical trials.	21-23 countries across northern and southern hemispheres	Healthy infants ≤12 months of age born between 29 wGA and full term	2 RCTs		Medically-attended RSV- LRTI, RSVH, AEs
					Nirsevimab (n=1963)	Placebo (n=980)	
**Riccò M et al. 2024**[42]	Pooled analysis	To systematically evaluate the efficacy and provide early estimates on the effectiveness of nirsevimab in infants and children in terms of avoiding RSVH, possibly providing guidance for medical and public health professionals.	At least 31 countries across northern and southern hemispheres	Children who had received at least one dose of nirsevimab for the prevention of RSV infection	5 RCTs, 7 observational studies and 1 health authority report		RSVH
					Nirsevimab (n=33,884)	No nirsevimab, placebo (n=9365)	
**Mahmud S et al. 2023**[43]	CEA	To provide a reference document for multidisciplinary country teams in LMICs that may be interested in using UNIVAC to explore the potential cost-effectiveness of strategies to prevent RSV disease in children aged <5 years.	133 LMICs	Children <5 years	Nirsevimab	Maternal vaccine, no intervention	Cost per DALY averted
**Koltai M et al. 2023**[44]	CEA	To provide updated estimates for the cost-effectiveness of MV or mAb-based public health interventions to prevent RSV disease in children under 5 years of age in two African countries, one lower middle income (Kenya) and the other upper middle income (South Africa).	Kenya and South Africa	Children <5 years	Nirsevimab	Maternal vaccine, no intervention	ICER per DALY averted
**Getaneh AM et al. 2023**[45]	CEA	To evaluate the cost-effectiveness of year-round RSV MI and mAb programs, as well as a seasonal RSV mAb program, and a seasonal mAb plus catchup program using the most recent country-specific data for six European countries.	Denmark, Finland, England, Scotland, Italy, and the Netherlands	Children <5 years	Nirsevimab	Maternal vaccine, no intervention	ICER, WTP/QALY

**Li X et al. 2023[46]**	CEA	To compare the outcomes of different available model-based analytical approaches designed to estimate the cost-effectiveness of RSV prevention in infancy and pregnancy using a standardised set of input parameters. Our objectives are (1) to understand the impact of model structure and parameterisation on model outcomes, (2) to investigate the robustness of model results to variations in assumptions, and (3) to generate insights for future RSV modelling efforts.	Hypothetical country in Europe	Hypothetical birth cohort	Nirsevimab	Maternal vaccine, no intervention	ICER/QALY
**Hodgson D et al. 2022[47]**	CEA	To evaluate the cost-effectiveness of Nirsevimab intervention programmes in England and Wales.	England and Wales	25 age groups (monthly up to 11 months of age, and then 1, 2, 3, 4, 5–9, 10– 14, 15–24, 25–34, 35–44, 45–54, 55–64, 65–74, 75+ years	Nirsevimab	Palivizumab	Purchasing price per dose for ICER/QALY
**Li X et al. 2022[48]**	CEA	To evaluate the health and economic burden of RSV disease and the cost-effectiveness of RSV disease prevention strategies, including both seasonal and year-round programs in Norwegian children under 5.	Norway	Children <5 years	Nirsevimab	Maternal vaccine, no intervention	ICER/QALY
**Liu D et al. 2021[49]**	CEA	To evaluate the cost-effectiveness of the administration of maternal immunisation (MI), infant mAb (IA) and paediatric immunisation (PI) as well as their combinations in eight Chinese cities.	China	Birth cohort	Nirsevimab	Maternal vaccine, no intervention	Threshold strategy cost
**Laufer RS et al. 2021[50]**	CEA	To estimate the likelihood of respiratory syncytial virus preventive interventions being cost-effective in Mali.	Mali	Birth cohort	Nirsevimab	Maternal vaccine, palivizumab, no intervention	ICER/DALY
**Hodgson D et al. 2024[51]**	CEA	To provide a head-to-head cost-effectiveness comparison of maternal vaccine and LAmAb programmes in England and Wales by integrating a transmission model calibrated to data on RSV incidence and clinical trial data into an economic evaluation. To calculate the optimal programme under a realistic range of purchase and administration costs.	England and Wales	25 age groups (monthly up to 11 months of age, and then 1, 2, 3, 4, 5–9, 10– 14, 15–24, 25–34, 35–44, 45–54, 55–64, 65–74, 75+ years	Nirsevimab	Maternal vaccine, palivizumab	Incremental NMB and Expected Value of Perfect Information under a range of cost of purchasing and administration

**Shoukat A et al. 2023**[52]	CEA	To conduct a comprehensive cost-effectiveness analysis of RSV infant and maternal immunisation strategies based on population demographics in the Canadian south (i.e. southern provinces of Canada excluding the three northern territories and Nunavik in Quebec)	Canada	Birth cohort	Nirsevimab	Maternal vaccine, no intervention	NMB and ICER/QALY
**Nourbakhsh S et al. 2021**[53]	CEA	To evaluate cost-effectiveness of immunisation programs with the next generation of RSV prophylactics, we developed an agent-based transmission model, parameterised with the estimated RSV disease burden in Nunavik.	Canada	Infants < 12 months of age who were healthy and born at full term or preterm/chronically ill	Nirsevimab	Maternal vaccine, palivizumab, no intervention	ICER/QALY
**Hutton D. 2023**[54]	CEA	To determine the cost effectiveness of nirsevimab.	USA	Infants < 7 months of age entering their first RSV season or high-risk infants 7-18 months old in their second RSV season	Nirsevimab	Palivizumab, no intervention	ICER/QALY
**Gebretekle GB et al. 2024**[55]	CEA	To conduct a model-based economic evaluation to evaluate the cost-effectiveness of multiple immunisation strategies for protecting Canadian infants against RSV disease under various scenarios.	Canada	Birth cohort	Nirsevimab	Maternal vaccine, palivizumab	ICER/QALY
**Kieffer A et al. 2022**[56]	Modelling analysis	To evaluate the health and cost outcomes associated with the use of nirsevimab against SoC in the prevention of medically-attended RSV-LRTIs in all infants in their first RSV season in the United States.	USA	Birth cohort	Nirsevimab	Palivizumab, no intervention	Medically-attended RSV-LRTI, RSVH, ICU admissions, mechanical ventilation cases averted and related cost savings
**Falavigna M et al. 2023**[57]	Modelling analysis	To model RSV-related hospitalisations and costs of the impact of nirsevimab on SUS population, compared to Standard of Practice.	Brazil	Birth cohort	Nirsevimab	Palivizumab, no intervention	RSV-LRTI and ICU admissions averted and related cost savings
**Falavigna M et al. 2023**[58]	Modelling analysis	To model RSV-related hospitalisations and costs of nirsevimab on Private Healthcare System, compared to Standard of Practice.	Brazil	Birth cohort	Nirsevimab	Palivizumab, no intervention	RSV-LRTI and ICU admissions averted and related cost savings
**Ren S et al. 2024**[59]	Modelling analysis	To model and seek the optimal seasonal mAb administration strategy for preventing RSVH.	China	Children < 2 years	Nirsevimab	Alternative year-round/seasonal administration strategies for nirsevimab	RSVH averted

**Ektare V et al. 2022**[60]	Modelling analysis	To develop a spreadsheet-based decision-tree model to evaluate the direct effects of RSV prevention interventions for infants (<1-year-olds) and high-risk children (<2-year-olds with CHD or CLD) on medically-attended RSV episodes and RSVH and recurrent wheezing due to RSV.	USA	Infants <12 months of age and high-risk children <24 months of age with CHD or CLD	Nirsevimab	Maternal vaccine, palivizumab, no intervention	Medically-attended RSV-LRTI, RSVH, ICU admissions, and recurrent wheeze cases averted
**Gomez GB et al. 2022**[61]	Modelling analysis	To assess the distribution of health benefits across insurance groups when accessing nirsevimab through alternative reimbursement pathways.	USA	Infants <12 months of age	Nirsevimab	Alternative reimbursement pathways for nirsevimab	Medically-attended RSV-LRTI and RSVH averted
**Maas BM et al. 2021**[62]	Modelling analysis	To better understand the relationship between MK-1654 dose and potential efficacy in humans, we report a model-based meta-analysis that quantifies the relationship between RSV serum neutralizing activity and clinically relevant endpoints in humans, including LRTI in infants. To predict clinical efficacy in infants across different dose-levels of MK-1654, which recently entered a phase 2b/3 clinical outcome trial in this population (NCT04767373).	USA	Healthy preterm and term infants < 8 months of age	Clesrovimab	Placebo	Relationship between RSV serum neutralizing activity and five RSV disease severity levels ranging from asymptomatic infection to severe (ICU admission)
**Prasad N et al. 2021**[63]	Modelling analysis	To estimate the impact of an RSV maternal vaccine and a seasonal infant mAb on RSVH, under varying levels of coverage and effectiveness, using a mathematical model fitted to population-based RSV hospital surveillance data from Auckland, NZ.	New Zealand	Children <24 months of age	Nirsevimab	Maternal vaccine, no intervention	RSVH averted
**Li Y et al. 2021**[64]	Modelling analysis	To assess the potential effect of different approaches for administration of monoclonal antibodies and maternal vaccination among LMICs by evaluating the annual and per-dose proportion of RSV-LRTI averted among infants younger than 6 months.	52 LMICs	Infants <6 months of age	Nirsevimab	Maternal vaccine	RSV-LRTI and RSVH averted
**Finelli L et al. 2020**[65]	Modelling analysis	To model the anticipated public health benefits of RSV LAmAb immunisation using the number needed to immunise.	USA	Infants <12 months of age	Nirsevimab	N/A	NNT to prevent RSVH and RSV outpatient visit

**Zheng Z et al. 2022[66]**	Modelling analysis	To estimate the potential impact of three RSV prevention strategies that aim to protect paediatric populations and to identify the key factors that affect vaccine impact.	USA	13 age categories: infants younger than 2 months, 2–3 months, 4–5 months, 6–7 months, 8–9 months, 10–11 months, 1 year, 2–4 years, 5–9 years, 10–19 years, 20–39 years, 40–59 years, and ≥60 years.	Nirsevimab	Maternal vaccine, live attenuated vaccine	RSVH averted
**Farid AT et al. 2022[67]**	Modelling analysis	To inform policies about possible immunisation, this literature-based model sought to estimate the adverse effects burden of administering nirsevimab, a passive protection against RSV in infants.	USA	Low-risk infants <12 months of age	Nirsevimab	N/A	DALY
**Mazagatos C et al. 2024[68]**	Modelling analysis	To estimate the impact of this nirsevimab in terms of prevented cases of RSVH in children < 1 year	Spain	Infants <12 months	Nirsevimab	No nirsevimab	RSVH averted
**Álvarez García FJ et al. 2024[69]**	Recommendations / guidelines	N/A	Spain	All infants/children eligible for nirsevimab	Nirsevimab	N/A	Recommendations for use
**Francisco L et al. 2023[70]**	Recommendations / guidelines	N/A	Spain	All infants/children eligible for nirsevimab	Nirsevimab	N/A	Recommendations for use
**Sánchez Luna M et al. 2023[71]**	Recommendations / guidelines	N/A	Spain	All infants/children eligible for nirsevimab	Nirsevimab	N/A	Recommendations for use
**O'Leary ST et al. 2023[72]**	Recommendations / guidelines	N/A	USA	All infants/children eligible for nirsevimab	Nirsevimab	N/A	Recommendations for use
**Jones JM et al. 2023[73]**	Recommendations / guidelines	N/A	USA	All infants/children eligible for nirsevimab	Nirsevimab	N/A	Recommendations for use
**American Academy of Pediatrics. 2024[74]**	Recommendations / guidelines	N/A	USA	All infants/children eligible for nirsevimab	Nirsevimab	N/A	Recommendations for use
**Medrano López C et al. 2024[75]**	Recommendations / guidelines	N/A	Spain	Children <24 months of age with CHD	Nirsevimab	N/A	Recommendations for use
**Society for Maternal-Fetal Medicine. 2024[76]**	Recommendations / guidelines	N/A	USA	All infants/children eligible for nirsevimab	Nirsevimab	N/A	Recommendations for use
**Fleming-Dutra KE et al. 2023[77]**	Recommendations / guidelines	N/A	USA	All infants/children eligible for nirsevimab	Nirsevimab	N/A	Recommendations for use
**Álvarez García FJ et al. 2023[78]**	Recommendations / guidelines	N/A	Spain	All infants/children eligible for nirsevimab	Nirsevimab	N/A	Recommendations for use
**Sparrow E et al. 2022[79]**	Recommendations / guidelines	N/A	Global (WHO)	All infants/children eligible for nirsevimab	Nirsevimab	N/A	Recommendations for use
**Alharbi AS et al. 2021[80]**	Recommendations / guidelines	N/A	Saudi Arabia	All infants/children eligible for nirsevimab	Nirsevimab	N/A	Recommendations for use

**Centers for Disease Control and Prevention. 2023**[81]	Recommendations / guidelines	N/A	USA	All infants/children eligible for nirsevimab	Nirsevimab	N/A	Recommendations for use
**National Advisory Committee on Immunization. 2024**[82]	Recommendations / guidelines	N/A	Canada	All infants/children eligible for nirsevimab	Nirsevimab	N/A	Recommendations for use
**Ministerio de Sanidad. 2024**[83,84]	Recommendations / guidelines	N/A	Spain	All infants/children eligible for nirsevimab	Nirsevimab	N/A	Recommendations for use
**Alharbi AS et al. 2024**[85]	Recommendations / guidelines	N/A	Saudi Arabia	All infants/children eligible for nirsevimab	Nirsevimab	N/A	Recommendations for use
**Debbag R et al. 2024**[86]	Recommendations / guidelines	N/A	Latin America	All infants/children eligible for nirsevimab	Nirsevimab	N/A	Recommendations for use
**UK Health Security Agency 2024**[87]	Recommendations / guidelines	N/A	UK	All infants/children eligible for nirsevimab	Nirsevimab	N/A	Recommendations for use
**CAVEI 2023**[88]	Recommendations / guidelines	N/A	Chile	All infants/children eligible for nirsevimab	Nirsevimab	N/A	Recommendations for use

ADA: anti-drug antibody; AE: adverse event; ARI: acute respiratory illness; CHD: congenital heart disease; CLD: chronic lung disease; DALY: disability-adjusted life year; ICER: incremental cost-effectiveness ratio; ICU: intensive care unit; IM: intramuscular; LAmAb: long-acting monoclonal antibody; LMIC: lower-middle income country; mAb: monoclonal antibody; N/A: not applicable; NAb: neutralising antibody; NMB: net monetary benefit; NNT: number needed to treat; PICU: paediatric intensive care unit; PK: pharmacokinetics; QALY: quality-adjusted life year RCT: randomised controlled trial; RSV: respiratory syncytial virus; RSVH: respiratory syncytial virus hospitalisation; RSV-LRTI: respiratory syncytial virus lower respiratory tract infection; SoC: standard of care; wGA: weeks' gestational age; WHO: World Health Organisation; WTP: willingness-to-pay threshold

Supplementary File 3: Risk of Bias Assessments

The risk of bias was assessed in each individual study using the most appropriate scoring tool.

For randomised clinical trials, version two of the Cochrane Collaboration risk of bias tool[5] consisting of multiple questions spanning five domains was used to assess bias. A judgement on the overall risk of bias was then made based upon the level of concern within each domain (low, some concern, high).

Study	Randomisation process	Deviations from intended interventions	Missing outcome data	Measurement of the outcome	Selection of reported results	Overall
**Drysdale SB et al. 2023[8]**	LOW	LOW	LOW	LOW	LOW	LOW
**Ahani B et al. 2023[9]**	LOW	LOW	LOW	LOW	LOW	LOW
**Domachowske JB et al. 2023[10]**	LOW	LOW	LOW	LOW	LOW	LOW
**Wilkins D et al. 2023[11]**	LOW	LOW	LOW	LOW	SOME CONCERNS	SOME CONCERNS
**Muller WJ et al. 2023[12]**	LOW	LOW	LOW	LOW	LOW	LOW
**Domachowske J et al. 2022[13]**	LOW	LOW	LOW	LOW	LOW	LOW
**Hammitt LL et al. 2022[14]**	LOW	LOW	LOW	LOW	LOW	LOW
**Griffin MP et al. 2020[15]**	LOW	LOW	LOW	LOW	LOW	LOW
**Domachowske JB et al. 2018[16]**	LOW	LOW	LOW	LOW	LOW	LOW
**Madhi 2024[17]**	LOW	LOW	LOW	LOW	LOW	LOW
**Arbetter D et al. 2024[21]**	LOW	LOW	LOW	LOW	SOME CONCERNS	SOME CONCERNS

For observational studies a modified version of the RTI Item Bank,[6] consisting of 13 questions was used to assess bias. The original RTI questions were reworded slightly to enable all YES answers to indicate a low risk of bias. The number of YES responses to applicable questions was then used to determine overall bias: ≤33% = HIGH bias; 34-67% = MODERATE bias; >67% = LOW

Study	Selection Bias		Performance Bias	Detection Bias			Attrition Bias		Selective Outcome Reporting		Confounding		Overall Assessment	Overall score
	Q1	Q2	Q3	Q4	Q5	Q6	Q7	Q8	Q9	Q10	Q11	Q12	Q13	
**López-Lacort M et al. 2024[23]**	YES	YES	YES	UNCLEAR	N/A	YES	YES	N/A	YES	NO	YES	NO	YES	8/11 (73%) LOW

**Ernst C et al. 2024[24]**	YES	YES	YES	UNCLEAR	N/A	YES	NO	N/A	YES	YES	YES	UNCLEAR	YES	8/11 (73%) LOW
**Martinón-Torres F et al. 2023[25]**	N/A	N/A	N/A	UNCLEAR	N/A	YES	N/A	N/A	YES	YES	YES	N/A	YES	5/6 (83%) LOW
**Consolati A et al. 2024[26]**	YES	YES	YES	UNCLEAR	N/A	YES	YES	N/A	YES	YES	YES	YES	YES	10/11 (91%) LOW
**Moline HL et al. 2024[27]**	YES	YES	YES	UNCLEAR	N/A	YES	YES	N/A	YES	NO	YES	YES	YES	9/11 (82%) LOW
**Paireau J et al. 2024[28]**	YES	YES	NO	UNCLEAR	N/A	YES	YES	N/A	YES	NO	YES	NO	YES	7/11 (64%) MODERATE
**Ares-Gómez S et al. 2024[29]**	YES	NO	YES	YES	N/A	YES	YES	N/A	YES	YES	YES	YES	YES	10/11 (91%) LOW
**Mallaha N et al. 2024[30]**	YES	NO	YES	YES	N/A	YES	YES	N/A	YES	YES	YES	YES	YES	10/11 (91%) LOW
**Ezpeleta G et al. 2024[31]**	YES	YES	YES	UNCLEAR	N/A	YES	YES	N/A	YES	YES	YES	NO	NO	8/11 (73% LOW
**Assad Z et al. 2024[32]**	YES	YES	YES	YES	N/A	YES	YES	N/A	YES	NO	YES	YES	YES	10/11 (91%) LOW
**Barbas Del Buey JF et al. 2024[33]**	YES	YES	YES	UNCLEAR	N/A	YES	YES	N/A	YES	NO	YES	NO	YES	8/11 (73% LOW
**Andina Martínez D et al. 2024[34]**	N/A	N/A	N/A	UNCLEAR	N/A	YES	N/A	N/A	YES	NO	YES	N/A	NO	3/6 (50%) MODERATE
**Moline HL et al.[35]**	YES	YES	YES	UNCLEAR	N/A	YES	YES	N/A	YES	NO	YES	YES	YES	9/11 (82%) LOW

For economic studies, the Quality of Health Economic Analyses tool[7] consisting of 16 individually marked questions was used to assess bias.

**Study**	**Objective**	**Perspective**	**Sources**		**Uncertainty**	**Study design, methodology and outcomes**								**Bias**	**Conclusions**	**Funding**	**Overall (/100)**
	**Q1 (/7)**	**Q2 (/4)**	**Q3 (/8)**	**Q4 (/1)**	**Q5 (/9)**	**Q6 (/6)**	**Q7 (/5)**	**Q8 (/7)**	**Q9 (/8)**	**Q10 (/6)**	**Q11 (/7)**	**Q12 (/8)**	**Q13 (/7)**	**Q14 (/6)**	**Q15 (/8)**	**Q16 (/3)**	
**Mahmud S et al. 2023[43]**	7	4	8	1	9	0	5	0	8	6	7	8	7	6	8	3	87

**Koltai M et al. 2023[44]**	7	0	8	1	9	6	5	0	8	0	7	8	7	6	8	3	83
**Getaneh AM et al. 2023[45]**	7	4	8	1	9	6	5	0	8	6	7	8	7	6	8	3	93
**Li X et al. 2023[46]**	7	0	8	1	9	6	5	0	8	0	7	8	7	6	8	3	83
**Hodgson D et al. 2022[47]**	7	0	8	1	9	6	0	7	8	0	7	8	7	6	8	3	85
**Li X et al. 2022[48]**	7	0	8	1	9	6	5	0	8	6	7	8	7	6	8	3	89
**Liu D et al. 2021[49]**	7	0	8	1	9	0	5	0	8	0	7	8	7	6	8	3	77
**Laufer RS et al. 2021[50]**	7	4	8	1	9	6	5	0	8	0	7	8	7	6	8	3	87
**Hodgson D et al. 2024[51]**	7	0	8	1	9	6	5	7	8	0	7	8	7	0	8	3	84
**Shoukat A et al. 2023[52]**	7	4	8	1	9	6	5	0	8	0	7	8	7	0	8	3	81
**Nourbakhsh S et al. 2021[53]**	7	0	8	1	0	6	5	0	8	0	7	8	7	0	8	3	68
**Hutton D. 2023[54]**	7	0	8	1	9	6	0	0	8	6	7	8	7	6	8	3	84
**Gebretekle GB et al. 2024[55]**	7	4	8	1	9	6	5	0	8	0	7	8	7	6	8	3	87

There were 44 references not quality assessed as they were abstracts/presentations (Dagan R et al. 2022[18], Domachowske JB et al. 2023[22],Zar HJ et al. 2024[19,20]), public health reports (Dirección Xeral de Saúde Pública. 2024[36]), pooled analyses (Wilkins D et al. 2023[37], Sun M et al. 2023[38], Simões EAF et al. 2023[39], Abram ME et al. 2022[40], Turalde-Mapili MWR et al. 2023[41], Riccò M et al. 2024[42]), modelling analyses other than cost-effectiveness (Kieffer A et al. 2022[56], Falavigna M et al. 2023[57], Falavigna M et al. 2023[58], Ren S et al. 2024[59], Ektare V et al. 2022[60], Gomez GB et al. 2022[61], Maas BM et al. 2021[62], Prasad N et al. 2021[63], Li Y et al. 2021[64], Finelli L et al. 2020[65], Zheng Z et al. 2022[66], Farid AT et al. 2022[67], Mazagatos C et al. 2024[68]) or recommendations/guidelines (Álvarez García FJ et al. 2024[69], Francisco L et al. 2023[70], Sánchez Luna M et al. 2023[71], O'Leary ST et al. 2023[72], Jones JM et al. 2023[73], American Academy of Pediatrics. 2024[74], Medrano López C et al. 2024[75], Society for Maternal-Fetal Medicine. 2024[76], Fleming-Dutra KE et al. 2023[77], Álvarez García FJ et al. 2023[78], Sparrow E et al. 2022[79], Alharbi AS et al. 2021[80], Centers for Disease Control and Prevention. 2023[81], National Advisory Committee on Immunization. 2024[82], Ministerio de Sanidad. 2024[83,84], Alharbi AS et al. 2024[85], Debbag R et al.[86], UK Health Secuirty Agency[87], CAVEI[88]).

References

[1] Li Y, Wang X, Blau DM, Caballero MT, Feikin DR, Gill CJ, et al. Global, regional, and national disease burden estimates of acute lower respiratory infections due to respiratory syncytial virus in children younger than 5 years in 2019: a systematic analysis. Lancet. 2022; 399(10340):2047–64.

[2] Reeves RM, van Wijhe M, Tong S, et al. Respiratory Syncytial Virus-Associated Hospital Admissions in Children Younger Than 5 Years in 7 European Countries Using Routinely Collected Datasets. J Infect Dis. 2020;222(Suppl 7):S599-S605.

[3] Moher D, Liberati A, Tetzlaff J, Altman DG, The PRISMA Group. Preferred Reporting Items for Systematic Reviews and Meta-Analyses: The PRISMA Statement. BMJ 2009;339:b2535.

[4] Paez A. Grey literature: An important resource in systematic reviews. J Evid Based Med. 2017. doi: 10.1111/jebm.12265. Online ahead of print.

[5] Sterne JAC, Savović J, Page MJ, et al. RoB 2: a revised tool for assessing risk of bias in randomised trials. BMJ 2019; 366: l4898.

[6] Viswanathan M, Berkman ND, Dryden DM, Hartling L. Assessing Risk of Bias and Confounding in Observational Studies of Interventions or Exposures: Further Development of the RTI Item Bank. Rockville (MD): Agency for Healthcare Research and Quality (US); August 2013.

[7] Ofman JJ, Sullivan SD, Neumann PJ, et al. Examining the value and quality of health economic analyses: implications of utilizing the QHES. J Manag Care Pharm. 2003;9(1):53-61.

[8] Drysdale SB, Cathie K, Flamein F, et al. Nirsevimab for Prevention of Hospitalizations Due to RSV in Infants. N Engl J Med. 2023;389(26):2425-2435.

[9] Ahani B, Tuffy KM, Aksyuk AA, et al. Molecular and phenotypic characteristics of RSV infections in infants during two nirsevimab randomized clinical trials. Nat Commun. 2023;14(1):4347.

[10] Domachowske JB, Chang Y, Atanasova V, et al. Safety of Re-dosing Nirsevimab Prior to RSV Season 2 in Children With Heart or Lung Disease. J Pediatric Infect Dis Soc. 2023;12(8):477-480.

[11] Wilkins D, Yuan Y, Chang Y, et al. Durability of neutralizing RSV antibodies following nirsevimab administration and elicitation of the natural immune response to RSV infection in infants. Nat Med. 2023;29(5):1172-1179.

[12] Muller WJ, Madhi SA, Seoane Nuñez B, et al. Nirsevimab for Prevention of RSV in Term and Late-Preterm Infants. N Engl J Med. 2023;388(16):1533-1534.

[13] Domachowske J, Madhi SA, Simões EAF, et al. Safety of Nirsevimab for RSV in Infants with Heart or Lung Disease or Prematurity. N Engl J Med. 2022;386(9):892-894.

[14] Hammitt LL, Dagan R, Yuan Y, et al. Nirsevimab for Prevention of RSV in Healthy Late-Preterm and Term Infants. N Engl J Med. 2022;386(9):837-846.

[15] Griffin MP, Yuan Y, Takas T, et al. Single-Dose Nirsevimab for Prevention of RSV in Preterm Infants. N Engl J Med. 2020;383(5):415-425.

[16] Domachowske JB, Khan AA, Esser MT, et al. Safety, Tolerability and Pharmacokinetics of MEDI8897, an Extended Half-life Single-dose Respiratory Syncytial Virus Prefusion F-targeting Monoclonal Antibody Administered as a Single Dose to Healthy Preterm Infants. Pediatr Infect Dis J. 2018;37(9):886-892.

[17] Madhi SA, Simões EAF, Acevedo A, et al. A Phase 1b/2a Single Ascending Dose Study of a Half-life Extended RSV Neutralizing Antibody, Clesrovimab, in Healthy Preterm and Full-term Infants. J Infect Dis. Published online November 27, 2024. doi:10.1093/infdis/jiae581

[18] Dagan R, Nunez BS, Cots MB, etal. Nirsevimab for the prevention of RSV disease in healthy late-preterm and term infants: follow-up through second RSV season. In: 12th International RSV Symposium; 2022.

[19] Zar HJ, Simoes EAF, Madhi SA, et al. A Phase 2b/3 Study to Evaluate the Efficacy and Safety of an Investigational Respiratory Syncytial Virus (RSV) Antibody, Clesrovimab, in Healthy Preterm and Full-Term Infants. Presented at IDWeek 2024, October 16–19, Los Angeles, California. Abstract #166.

[20] Zar HJ, Bont LJ, Manzoni P, et al. Phase 3, Randomized, Controlled Trial Evaluating Safety, Efficacy and Pharmacokinetics of Clesrovimab in Infants and Children at Increased Risk for Severe Respiratory Syncytial Virus Disease. Presented at IDWeek 2024, October 16–19, Los Angeles, California. Abstract #167.

[21] Arbetter D, Gopalakrishnan V, Aksyuk AA, et al. Lower respiratory tract infections following respiratory syncytial virus monoclonal antibody nirsevimab immunization versus placebo: Analysis from a Phase 3 randomized clinical trial (MELODY). Clin Infect Dis. Published online December 4, 2024. doi:10.1093/cid/ciae596

[22] Domachowske JB, Wahlby Hamren U, Basavaraju B, et al. Safety, Tolerability, and Pharmacokinetics of Nirsevimab for the Prevention of RSV Disease in Immunocompromised Children Aged <=24 Months: Music, an Open Label, Phase 2 Trial. Blood. 2023;142(Supplement 1):1173.

[23] López-Lacort M, Muñoz-Quiles C, Mira-Iglesias A, et al. Early estimates of nirsevimab immunoprophylaxis effectiveness against hospital admission for respiratory syncytial virus lower respiratory tract infections in infants, Spain, October 2023 to January 2024. Euro Surveill. 2024;29(6):2400046.
[24] Ernst C, Bejko D, Gaasch L, et al. Impact of nirsevimab prophylaxis on paediatric respiratory syncytial virus (RSV)-related hospitalisations during the initial 2023/24 season in Luxembourg. Euro Surveill. 2024;29(4):2400033.
[25] Martinón-Torres F, Mirás-Carballal S, Durán-Parrondo C. Early lessons from the implementation of universal respiratory syncytial virus prophylaxis in infants with long-acting monoclonal antibodies, Galicia, Spain, September and October 2023. Euro Surveill. 2023;28(49):2300606.
[26] Consolati A, Farinelli M, Serravalle P, et al. Safety and Efficacy of Nirsevimab in a Universal Prevention Program of Respiratory Syncytial Virus Bronchiolitis in Newborns and Infants in the First Year of Life in the Valle d'Aosta Region, Italy, in the 2023-2024 Epidemic Season. Vaccines (Basel). 2024;12(5):549.
[27] Moline HL, Tannis A, Toepfer AP, et al. Early Estimate of Nirsevimab Effectiveness for Prevention of Respiratory Syncytial Virus-Associated Hospitalization Among Infants Entering Their First Respiratory Syncytial Virus Season - New Vaccine Surveillance Network, October 2023-February 2024. MMWR Morb Mortal Wkly Rep. 2024;73(9):209-214.
[28] Paireau J, Durand C, Raimbault S, et al. Nirsevimab Effectiveness Against Cases of Respiratory Syncytial Virus Bronchiolitis Hospitalised in Paediatric Intensive Care Units in France, September 2023-January 2024. Influenza Other Respir Viruses. 2024;18(6):e13311.
[29] Ares-Gómez S, Mallah N, Santiago-Pérez MI, et al. Effectiveness and impact of universal prophylaxis with nirsevimab in infants against hospitalisation for respiratory syncytial virus in Galicia, Spain: initial results of a population-based longitudinal study [published correction appears in Lancet Infect Dis. 2024 Jul;24(7):e419]. Lancet Infect Dis. Published online April 30, 2024. doi:10.1016/S1473-3099(24)00215-9
[30] Mallaha N, Jacobo Pardo-Secoa J, Pérez-Martínezj O, Durán-Parrondok C, Martinón-Torresa F on behalf of the NIRSE-GAL study group. Full 2023–24 season results of universal prophylaxis with nirsevimab in Galicia, Spain: the NIRSE-GAL study. Lancet Infect Dis. Published online December 11, 2024 https://doi.org/10.1016/S1473-3099(24)00811-9.
[31] Ezpeleta G, Navascués A, Viguria N, et al. Effectiveness of Nirsevimab Immunoprophylaxis Administered at Birth to Prevent Infant Hospitalisation for Respiratory Syncytial Virus Infection: A Population-Based Cohort Study. Vaccines (Basel). 2024;12(4):383.
[32] Assad Z, Romain AS, Aupiais C, et al. Nirsevimab and Hospitalization for RSV Bronchiolitis. N Engl J Med. 2024;391(2):144-154.
[33] Barbas Del Buey JF, Íñigo Martínez J, Gutiérrez Rodríguez MÁ, et al. The effectiveness of nirsevimab in reducing the burden of disease due to respiratory syncytial virus (RSV) infection over time in the Madrid region (Spain): a prospective population-based cohort study. Front Public Health. 2024;12:1441786.
[34] Andina Martínez D, Claret Teruel G, Gijón Mediavilla M, et al. Nirsevimab and Acute Bronchiolitis Episodes in Pediatric Emergency Departments. Pediatrics. 2024;154(4):e2024066584.
[35] Moline HL, Toepfer AP, Tannis A, et al. Respiratory Syncytial Virus Disease Burden and Nirsevimab Effectiveness in Young Children From 2023-2024. JAMA Pediatr. Published online December 9, 2024. doi:10.1001/jamapediatrics.2024.5572
[36] Dirección Xeral de Saúde Pública. Follow-Up Report on Immunization with Nirsevimab in Galicia - Data up to week 9, 2024 (03-03-2024); Dirección Xeral de Saúde Pública: Santiago de Compostela, Spain, 2024.
[37] Wilkins D, Langedijk AC, Lebbink RJ, et al. Nirsevimab binding-site conservation in respiratory syncytial virus fusion glycoprotein worldwide between 1956 and 2021: an analysis of observational study sequencing. Lancet Infect Dis. 2023;23(7):856-866.
[38] Sun M, Lai H, Na F, et al. Monoclonal Antibody for the Prevention of Respiratory Syncytial Virus in Infants and Children: A Systematic Review and Network Meta-analysis. JAMA Netw Open. 2023;6(2):e230023.
[39] Simões EAF, Madhi SA, Muller WJ, et al. Efficacy of nirsevimab against respiratory syncytial virus lower respiratory tract infections in preterm and term infants, and pharmacokinetic extrapolation to infants with congenital heart disease and chronic lung disease: a pooled analysis of randomised controlled trials. Lancet Child Adolesc Health. 2023;7(3):180-189.
[40] Abram ME, Ahani B, Tabor DE, et al. 94. Pooled analysis of nirsevimab resistance through 150 days post dose in preterm and term infants. Open Forum Infect Dis. 2022;9(Supplement 2):ofac492.019.
[41] Turalde-Mapili MWR, Mapili JAL, Turalde CWR, Pagcatipunan MR. The efficacy and safety of nirsevimab for the prevention of RSV infection among infants: A systematic review and meta-analysis. Front Pediatr. 2023;11:1132740.
[42] Riccò M, Cascio A, Corrado S, et al. Impact of Nirsevimab Immunization on Pediatric Hospitalization Rates: A Systematic Review and Meta-Analysis (2024). Vaccines (Basel). 2024;12(6):640.

[43] Mahmud S, Baral R, Sanderson C, et al. Cost-effectiveness of pharmaceutical strategies to prevent respiratory syncytial virus disease in young children: a decision-support model for use in low-income and middle-income countries. BMC Med. 2023;21(1):138.
[44] Koltai M, Moyes J, Nyawanda B, et al. Estimating the cost-effectiveness of maternal vaccination and monoclonal antibodies for respiratory syncytial virus in Kenya and South Africa. BMC Med. 2023;21(1):120.
[45] Getaneh AM, Li X, Mao Z, et al. Cost-effectiveness of monoclonal antibody and maternal immunization against respiratory syncytial virus (RSV) in infants: Evaluation for six European countries. Vaccine. 2023;41(9):1623-1631.
[46] Li X, Hodgson D, Flaig J, et al. Cost-Effectiveness of Respiratory Syncytial Virus Preventive Interventions in Children: A Model Comparison Study. Value Health. 2023;26(4):508-518.
[47] Hodgson D, Koltai M, Krauer F, Flasche S, Jit M, Atkins KE. Optimal Respiratory Syncytial Virus intervention programmes using Nirsevimab in England and Wales. Vaccine. 2022;40(49):7151-7157.
[48] Li X, Bilcke J, Vázquez Fernández L, et al. Cost-effectiveness of Respiratory Syncytial Virus Disease Prevention Strategies: Maternal Vaccine Versus Seasonal or Year-Round Monoclonal Antibody Program in Norwegian Children. J Infect Dis. 2022;226(Suppl 1):S95-S101.
[49] Liu D, Leung K, Jit M, Wu JT. Cost-effectiveness of strategies for preventing paediatric lower respiratory infections associated with respiratory syncytial virus in eight Chinese cities. Vaccine. 2021;39(39):5490-5498.
[50] Laufer RS, Driscoll AJ, Baral R, et al. Cost-effectiveness of infant respiratory syncytial virus preventive interventions in Mali: A modeling study to inform policy and investment decisions. Vaccine. 2021;39(35):5037-5045.
[51] Hodgson D, Wilkins N, van Leeuwen E, et al. Protecting infants against RSV disease: an impact and cost-effectiveness comparison of long-acting monoclonal antibodies and maternal vaccination. Lancet Reg Health Eur. 2024;38:100829.
[52] Shoukat A, Abdollahi E, Galvani AP, et al. Cost-effectiveness analysis of nirsevimab and maternal RSVpreF vaccine strategies for prevention of Respiratory Syncytial Virus disease among infants in Canada: a simulation study. Lancet Reg Health Am. 2023;28:100629.
[53] Nourbakhsh S, Shoukat A, Zhang K, et al. Effectiveness and cost-effectiveness of RSV infant and maternal immunization programs: A case study of Nunavik, Canada. EClinicalMedicine. 2021;41:101141.
[54] Hutton D. Economic Analysis of Nirsevimab in Pediatric Populations. Updated Feb 2023 ACIP presentation. https://www.cdc.gov/vaccines/acip/meetings/downloads/slides-2023-08-3/02-RSV-jones-508.pdf (Accessed October 2024)
[55] Gebretekle GB, Yeung MW, Ximenes R, et al. Cost-effectiveness of RSVpreF vaccine and nirsevimab for the prevention of respiratory syncytial virus disease in Canadian infants. Vaccine. 2024:126164.
[56] Kieffer A, Beuvelet M, Sardesai A, et al. Expected Impact of Universal Immunization With Nirsevimab Against RSV-Related Outcomes and Costs Among All US Infants in Their First RSV Season: A Static Model. J Infect Dis. 2022;226(Suppl 2):S282-S292.
[57] Falavigna M, Watanabe SF, Santoro J, et al. Modelled Impact of Nirsevimab for All Infants in the Prevention of Respiratory Syncytial Virus (RSV): Related Hospitalizations and Its Predicted Cost to the Brazilian Public Healthcare System. Value in Health. 2023;26(12 Supplement):S26.
[58] Falavigna M, Watanabe SF, Santoro J, et al. CO103 Modelled Impact of Nirsevimab for All Infants in Preventing Respiratory Syncytial Virus (RSV): Related Hospitalizations and Costs in the Brazilian Private Healthcare System. Value in Health. 2023;26(12 Supplement):S33.
[59] Ren S, Chen Q, Zhang Y, et al. Modeling the optimal seasonal monoclonal antibody administration strategy for respiratory syncytial virus (RSV) prevention based on age-season specific hospitalization rate of RSV in Suzhou, China, 2016-2022. Vaccine. 2024;42(2):352-361.
[60] Ektare V, Lang J, Choi Y, Finelli L. The clinical impact of multiple prevention strategies for respiratory syncytial virus infections in infants and high-risk toddlers in the United States. Vaccine. 2022;40(42):6064-6073.
[61] Gomez GB, Nelson CB, Rizzo C, Shepard DS, Chaves SS. Inequalities in Health Impact of Alternative Reimbursement Pathways for Nirsevimab in the United States. J Infect Dis. 2022;226(Suppl 2):S293-S299.
[62] Maas BM, Lommerse J, Plock N, et al. Forward and reverse translational approaches to predict efficacy of neutralizing respiratory syncytial virus (RSV) antibody prophylaxis. EBioMedicine. 2021;73:103651.
[63] Prasad N, Read JM, Jewell C, et al. Modelling the impact of respiratory syncytial virus (RSV) vaccine and immunoprophylaxis strategies in New Zealand. Vaccine. 2021;39(31):4383-4390.

[64] Li Y, Hodgson D, Wang X, Atkins KE, Feikin DR, Nair H. Respiratory syncytial virus seasonality and prevention strategy planning for passive immunisation of infants in low-income and middle-income countries: a modelling study. Lancet Infect Dis. 2021;21(9):1303-1312.
[65] Finelli L, Choi Y, Goldstein E. Number needed to immunize to prevent RSV with extended half-life monoclonal antibody. Vaccine. 2020;38(34):5474-5479.
[66] Zheng Z, Weinberger DM, Pitzer VE. Predicted effectiveness of vaccines and extended half-life monoclonal antibodies against RSV hospitalizations in children. npj Vaccines. 2022;7(1):127.
[67] Farid AT, Hariharan D, Shepard DS. EPH72 Potential Adverse Effects of Passive Immunization Against Respiratory Syncytial Virus (RSV) in Low-Risk Infants in the United States. Value in Health. 2022;25(7 Supplement):S448.
[68] Mazagatos C, Mendioroz J, Rumayor MB, et al. Estimated Impact of Nirsevimab on the Incidence of Respiratory Syncytial Virus Infections Requiring Hospital Admission in Children < 1 Year, Weeks 40, 2023, to 8, 2024, Spain. Influenza Other Respir Viruses. 2024 May;18(5):e13294.
[69] Álvarez García FJ, Iofrío de Arce A, Álvarez Aldeán J, et al. Immunisation schedule of the Spanish Association of Pediatrics: 2024 recommendations. An Pediatr (Engl Ed). 2024;100(1):34-45.
[70] Francisco L, Cruz-Cañete M, Pérez C, et al. Nirsevimab for the prevention of respiratory syncytial virus disease in children. Statement of the Spanish Society of Paediatric Infectious Disease (SEIP). An Pediatr (Engl Ed). 2023;99(4):257-263.
[71] Sánchez Luna M, Fernández Colomer B, Couce Pico ML; en representación de la Junta Directiva de la Sociedad española de Neonatología SENEO Comisión de Infecciones SENEO y Comisión de Estándares de SENEO. Recommendations of the Spanish Society of Neonatology for the prevention of severe respiratory syncytial virus infections with nirsevimab, for the 2023-2024 season. An Pediatr (Engl Ed). 2023;99(4):264-265.
[72] O'Leary ST, Yonts AB, Gaviria-Agudelo C, Kimberlin DW, Paulsen GC. Summer 2023 ACIP Update: RSV Prevention and Updated Recommendations on Other Vaccines. Pediatrics. 2023;152(5):e2023063955.
[73] Jones JM, Fleming-Dutra KE, Prill MM, et al. Use of Nirsevimab for the Prevention of Respiratory Syncytial Virus Disease Among Infants and Young Children: Recommendations of the Advisory Committee on Immunization Practices - United States, 2023. MMWR Morb Mortal Wkly Rep. 2023;72(34):920-925.
[74] American Academy of Pediatrics. AAP Recommendations for the Prevention of RSV Disease in Infants and Children. 2024. https://publications.aap.org/redbook/resources/25379/AAP-Recommendations-for-the-Prevention-of-RSV (Accessed October 2024)
[75] Medrano López C, Centeno Malfaz F, Garcés Sánchez M; en representación de la Sociedad Española de Cardiología Pediátrica y Cardiopatías Congénitas; el grupo de cardiología clínica de SECPCC y el Comité Asesor de Vacunas de la Asociación Española de Pediatría. Recommendations of the Spanish Society of Pediatric Cardiology and Congenital Heart Diseases for the prevention of respiratory syncytial virus infections with nirsevimab in pediatric cardiology. An Pediatr (Engl Ed). 2024;100(2):148-150.
[76] Society for Maternal-Fetal Medicine. Joseph NT, Kuller JA, Louis JM, Hughes BL. Society for Maternal-Fetal Medicine Statement: Clinical considerations for the prevention of respiratory syncytial virus disease in infants. Am J Obstet Gynecol. 2024;230(2):B41-B49.
[77] Fleming-Dutra KE, Jones JM, Roper LE, Prill MM, Ortega-Sanchez IR, Moulia DL, Wallace M, Godfrey M, Broder KR, Tepper NK, Brooks O, Sánchez PJ, Kotton CN, Mahon BE, Long SS, McMorrow ML. Use of the Pfizer Respiratory Syncytial Virus Vaccine During Pregnancy for the Prevention of Respiratory Syncytial Virus-Associated Lower Respiratory Tract Disease in Infants: Recommendations of the Advisory Committee on Immunization Practices - United States, 2023. MMWR Morb Mortal Wkly Rep. 2023 Oct 13;72(41):1115-1122.
[78] Álvarez García FJ, Cilleruelo Ortega MJ, Álvarez Aldeán J, Garcés-Sánchez M, Garrote Llanos E, Iofrío de Arce A, Montesdeoca Melián A, Navarro Gómez ML, Pineda Solas V, Rivero Calle I, Ruiz-Contreras J, Serrano Marchuet P; en representación del Comité Asesor de Vacunas de la Asociación Española de Pediatría (CAV-AEP). Immunisation schedule of the Spanish Association of Paediatrics: 2023 Recommendations. An Pediatr (Engl Ed). 2023 Jan;98(1):58.e1-58.e10.
[79] Sparrow E, Adetifa I, Chaiyakunapruk N, Cherian T, Fell DB, Graham BS, Innis B, Kaslow DC, Karron RA, Nair H, Neuzil KM, Saha S, Smith PG, Srikantiah P, Were F, Zar HJ, Feikin D. WHO preferred product characteristics for monoclonal antibodies for passive immunization against respiratory syncytial virus (RSV) disease in infants - Key considerations for global use. Vaccine. 2022 Jun 9;40(26):3506-3510.
[80] Alharbi AS, Alzahrani M, Alodayani AN, Alhindi MY, Alharbi S, Alnemri A. Saudi experts' recommendation for RSV prophylaxis in the era of COVID-19: Consensus from the Saudi Pediatric Pulmonology Association. Saudi Med J. 2021;42(4):355-362.

[81] Centers for Disease Control and Prevention. Limited Availability of Nirsevimab in the United States—Interim CDC Recommendations to Protect Infants from Respiratory Syncytial Virus (RSV) during the 2023–2024 Respiratory Virus Season. 2023. https://emergency.cdc.gov/han/2023/han00499.asp (Accessed October 2024)

[82] National Advisory Committee on Immunization. An Advisory Committee Statement (ACS) National Advisory Committee on Immunization (NACI): Statement on the prevention of respiratory syncytial virus (RSV) disease in infants. 2024. https://www.canada.ca/content/dam/phac-aspc/documents/services/publications/vaccines-immunization/national-advisory-committee-immunization-statement-prevention-respiratory-syncytial-virus-disease-infants/naci-statement-2024-05-17.pdf (Accessed October 2024)

[83] Ministerio de Sanidad. Recomendaciones de utilización de nirsevimab para la temporada 2024-2025 en España. Available at: https://www.sanidad.gob.es/en/areas/promocionPrevencion/vacunaciones/comoTrabajamos/docs/Nirsevimab.pdf (Accessed October 2024)

[84] Ministerio de Sanidad. Actualización de recomendaciones de utilización de nirsevimab para la temporada 2024-2025 en España. Available at: https://www.sanidad.gob.es/areas/promocionPrevencion/vacunaciones/comoTrabajamos/docs/NirsevimabActualizacion.pdf (Accessed October 2024)

[85] Alharbi AS, Al-Hindi, MY, Alqwaiee M, et al. Saudi Initiative of Bronchiolitis Diagnosis, Management, and Prevention 2024 updated consensus on the prevention of respiratory syncytial virus. Ann Thorac Med. 2024;19(3):190-20.0

[86] Debbag R, Ávila-Agüero ML, Brea J, et al. Confronting the challenge: a regional perspective by the Latin American pediatric infectious diseases society (SLIPE) expert group on respiratory syncytial virus—tackling the burden of disease and implementing preventive solutions. Front Pediatr. 2024;12:1386082.

[87] UK Health Security Agency. Respiratory syncytial virus: the green book, chapter 27a. 2024. Available at: https://assets.publishing.service.gov.uk/media/669a5e37ab418ab05559290d/Green-book-chapter-27a-RSV-18_7_24.pdf (Accessed October 2024)

[88] Advisory Committee on Vaccines and Vaccination Strategies (CAVEI). CAVEI recommendation on incorporation of a monoclonal antibody for passive immunization against respiratory syncytial virus in infants in the National Immunization Program. Rev Chil Infectol. 2023;40(6): 657-664.
